# Histone demethylase Lsd1 is required for the differentiation of neural cells in *Nematostella vectensis*

**DOI:** 10.1038/s41467-022-28107-z

**Published:** 2022-01-24

**Authors:** James M. Gahan, Ian U. Kouzel, Kamilla Ormevik Jansen, Pawel Burkhardt, Fabian Rentzsch

**Affiliations:** 1 Sars International Centre for Marine Molecular Biology, University of Bergen, Thormøhlensgt 55, 5006 Bergen, Norway; 2 Department for Biological Sciences, University of Bergen, Thormøhlensgt 53, 5006 Bergen, Norway

**Keywords:** Evolutionary developmental biology, Neurogenesis

## Abstract

Chromatin regulation is a key process in development but its contribution to the evolution of animals is largely unexplored. Chromatin is regulated by a diverse set of proteins, which themselves are tightly regulated in a cell/tissue-specific manner. Using the cnidarian *Nematostella vectensis* as a basal metazoan model, we explore the function of one such chromatin regulator, Lysine specific demethylase 1 (Lsd1). We generated an endogenously tagged allele and show that NvLsd1 expression is developmentally regulated and higher in differentiated neural cells than their progenitors. We further show, using a CRISPR/Cas9 generated mutant that loss of *NvLsd1* leads to developmental abnormalities. This includes the almost complete loss of differentiated cnidocytes, cnidarian-specific neural cells, as a result of a cell-autonomous requirement for *NvLsd1*. Together this suggests that the integration of chromatin modifying proteins into developmental regulation predates the split of the cnidarian and bilaterian lineages and constitutes an ancient feature of animal development.

## Abstract (web summary)

The evolutionary point where chromatin modifier function integrated into regulation of specific cell types is unclear. In the cnidarian Nematostella vectensis, the authors here show that lysine specific demethylase Lsd1 is developmentally regulated and required for normal development including cnidocyte differentiation.

## Introduction

The evolution of animals and other multicellular organisms was accompanied by the emergence of the processes of cell-type specification and differentiation. These processes are tightly controlled in order to produce adults containing, in some cases, billions of cells and hundreds of different cell types originating from a single-celled embryo. The regulation of cell differentiation is largely governed on the transcriptional level. This has classically been viewed as the purview of DNA binding transcription factors that regulate sets of genes in a spatially and temporally restricted manner to drive cell identity and differentiation^1^. In recent years the role of chromatin modifications in this process has become more appreciated^2–4^. In particular, it is now clear that chromatin modifiers are not static, permissive actors in the process of cell differentiation but rather play an active role^4^. Indeed, chromatin modifiers such as those involved in histone lysine methylation and demethylation often play cell or tissue-specific roles^5,6^. Such chromatin modifiers can themselves be regulated, at the level of expression, splicing, localization, and activity to allow for differential functions in different cells or tissues. Transcriptional regulation by chromatin modifiers is present in unicellular eukaryotes, but it is currently not known at what point in the evolution of animals this function became integrated into the regulatory programs that control the differentiation of specific cell types. Here, we address this question by analyzing the role of the conserved chromatin regulator Lsd1/KDM1A in the development of an early-branching metazoan, the sea anemone *Nematostella vectensis*.

Lsd1 acts, in the majority of cases, to remove mono- or di-methylation on lysine 4 of histone H3 (H3K4me1/me2)^7^. It does so in a complex with Histone Deacetylase 1/2 (HDAC1/2) and Corepressor of REST (CoREST) in order to repress target genes^8–14^. In other cases, however, it has been shown that Lsd1 is involved in demethylation of H3K9 or H4K20 and can act as a transcriptional co-activator^15–18^. Loss of Lsd1 in all cells leads to early lethality in mice^15^, whereas tissue-specific manipulations of Lsd1 function revealed roles in the development and homeostasis of several organs and cell types^15,19–27^. Lsd1 has been shown to be particularly important in the nervous system where it regulates neurogenesis at several levels. In rodents, Lsd1 levels are higher in neural stem/progenitor cells and decrease as differentiation progresses^28–30^. Lsd1 plays roles in the maintenance of progenitor identity and proliferation and the decrease in Lsd1 levels is required for neural differentiation^28–30^. Lsd1 is also important in the later stages of neural differentiation, for example for cortical migration of differentiating neurons in mice^31^ and for the terminal differentiation of rod photoreceptors^32^. In humans, *LSD1* mutations have been linked to a developmental disorder that includes severe cognitive impairment^33,34^. In contrast to rodents, however, LSD1 levels remain largely unchanged or increase slightly during neural differentiation^18,35^. In *Drosophila melanogaster*, loss of Lsd1 leads to a range of developmental abnormalities without being lethal^36^. In *Caenorhabditis elegans*, the Lsd1 homolog SPR5 is required to erase H3K4me2 in primordial germ cells and its loss leads to a transgenerational loss of fertility^37^.

**Fig. 1:**
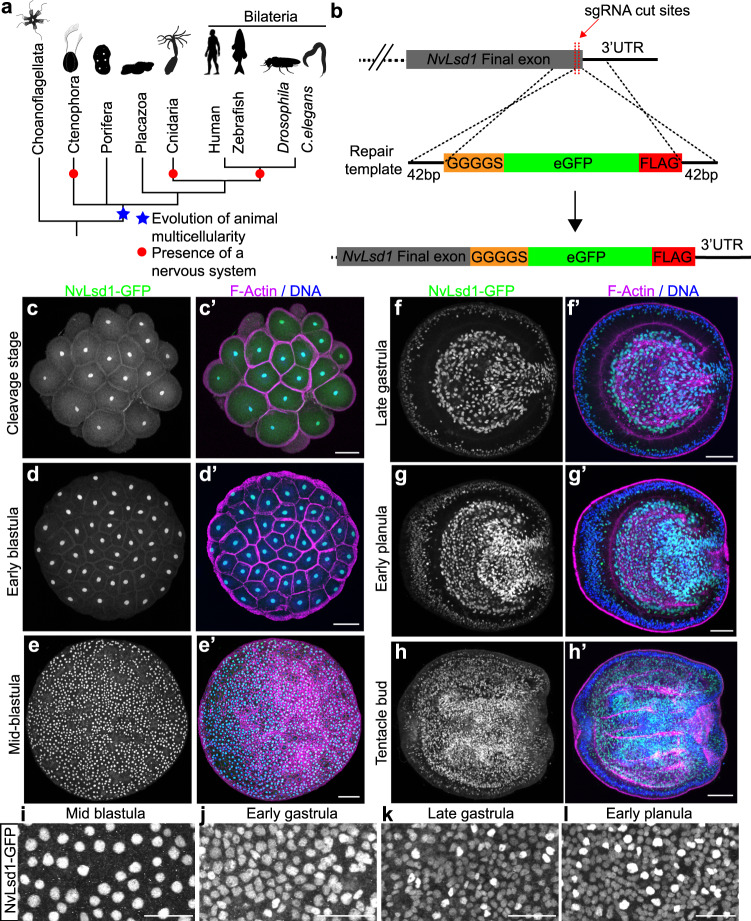
NvLsd1 is ubiquitously expressed but developmentally regulated. **a** Reduced phylogeny showing the position of the four non-bilaterian animal lineages with respect to the Bilateria. The evolution of animal multicellularity is annotated by a blue star and the lineages possessing a nervous system are marked with a red circle. Phylogeny based on^39^, animal silhouettes are from http://phylopic.org/. **b** Schematic showing the strategy for generating the *NvLsd1*^*GFP*^ knock-in allele by CRISPR-Cas9-mediated homologous recombination. **c**–**h** Confocal images of immunofluorescence staining on *NvLsd1*^*GFP*^ animals. Stage is shown on the left. Oral is shown to the right in (f–h). NvLsd1-GFP is shown in green, DNA in blue and F-Actin in magenta. **f**–**h** show mid-lateral views. **i**–**l** Close ups of NvLsd1-GFP in the ectoderm at different stages. Scale bars: 50 μm (**c**–**h**), 20 μm (**i**–**l**). Images shown are representative samples from at least two independent replicates with >10 animals per replicate.

Despite the importance of chromatin regulation during development, it is only well understood in a small number of “model” systems. All of these models belong to the Bilateria, the clade of animals containing most animal phyla (Fig. 1a). In order to understand how these processes evolved it is important to look at other, earlier diverging groups of animals. *Nematostella vectensis*, the starlet sea anemone, belongs to the sister group to the Bilateria, the Cnidaria (Fig. 1a), which separated from the lineage leading to bilaterians approximately 600 million years ago^38,39^. This key phylogenetic position along with the expanding molecular and genetic toolkit available, make *Nematostella* a useful system in which to address developmental questions in detail while also revealing evolutionary aspects of developmental processes^40^. Descriptive work at the whole organism level in *Nematostella* has shown that active chromatin modifications display similar patterns genome wide to those seen in other animals^41^ and studies in other cnidarians suggest roles for histone acetylation in development^42,43^.

*Nematostella* development proceeds via a hollow blastula stage and gastrulation by invagination to form a free-swimming planula larva. About one week after fertilization, the planula settles and develops into a sessile primary polyp that uses a ring of tentacles surrounding the only body opening to catch prey^44–46^ (Supplementary Fig. 1a). As for all cnidarians, *Nematostella* tissue has only two germ layers, which we here call ectoderm and endomesoderm (orange in Supplementary Fig. 1a). The *Nematostella* nervous system consists of a nerve net, thought to be an ancestral characteristic of animal nervous systems^47^. As in other cnidarians, there are three major, morphological cell types in the *Nematostella* nervous system; sensory cells, ganglionic neurons, and cnidocytes (cnidarian-specific neural cells also known as stinging cells)^48,49^. Recent years have seen advances in the understanding of the development of this nervous system, with a focus on transcription factors and signaling pathways^50–62^. How chromatin is regulated during neurogenesis and how chromatin modifiers are integrated with cell differentiation in *Nematostella* is unknown.

Here we use Lsd1 as a paradigm for a developmentally regulated chromatin modifier to examine chromatin-based regulation of development and cell differentiation in *Nematostella*. Employing CRISPR/Cas9-mediated genome editing, we generated an endogenously tagged allele and show that NvLsd1 is expressed at elevated levels in differentiated neural cells. Morphological and molecular analyses of a loss-of-function mutant identify defects in the differentiation of cnidocytes that can be rescued by cnidocyte-specific re-expression of *NvLsd1*. These results suggest that *NvLsd1* has an ancient function in neural cell differentiation.

## Results

### NvLsd1 is developmentally regulated

**Fig. 2:**
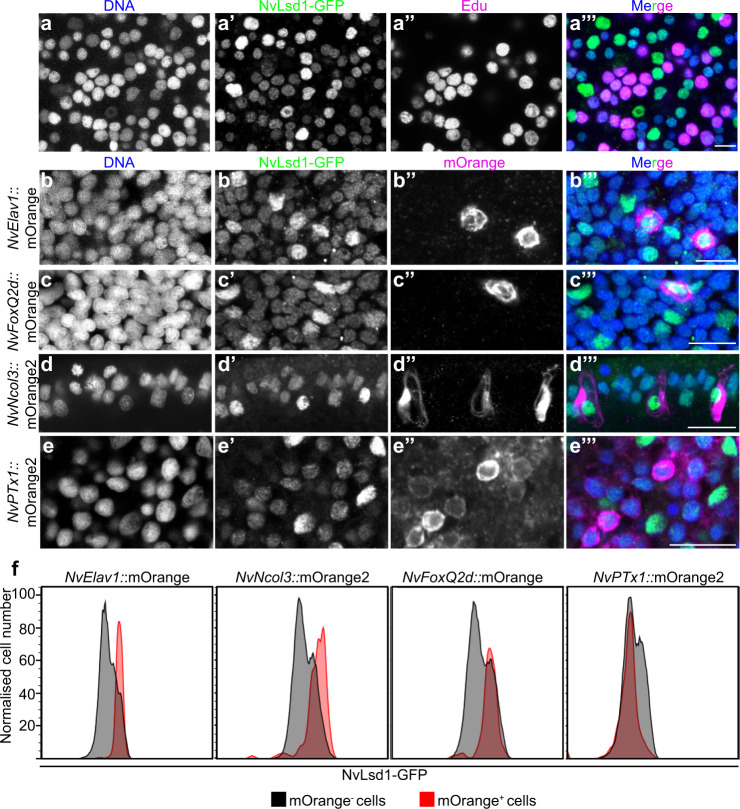
NvLsd1 is highly expressed in differentiated neural cells. **a** Confocal images showing close up of the ectoderm of *NvLsd1*^*GFP*^ late gastrula pulse labeled with EdU for 4 h stained with anti-GFP antibody (green) and Click-IT EdU (Magenta). DNA is shown in blue. **b**–**e** Confocal images of immunofluorescence staining on late planula of *NvLsd1*^*GFP*^ animals crossed with *NvElav1*::mOrange (**b**), *NvFoxQ2d*::mOrange (**c**), *NvNcol3*::mOrange2 (**d**) and mid-planula crossed with *NvPTx1*::mOrange2 (**e**) transgenics. DNA is shown in blue, GFP in green and mOrange in magenta. Images shown are representative samples from at least two independent replicates with >10 animals per replicate. **f** Histograms of flow cytometry data on NvLsd1^*GFP*^ animals crossed to the indicated transgenic lines. The *X*-axis shows the GFP fluorescence levels (arbitrary units), the *Y*-axis shows the cell number normalized to mode to correct for the different numbers of cells in the mOrange^+^ and mOrange^-^ gates. Scale bars: 20 μm.

Lsd/KDM1 genes are present throughout eukaryotes and fall into two clades; Lsd1/KDM1A and Lsd2/KDM1B^63^. Previous analyses have shown that the split into these two clades occurred at the base of the eukaryotes, and we found that representatives of the basal metazoan lineages Ctenophora, Porifera, and Cnidaria also possess these two paralogs (Supplementary Fig. 1b, Supplementary Data 1 and 2). Hereafter, we refer to the previously identified, highly conserved *Nematostella* homolog of Lsd1^63^ as *NvLsd1* (Supplementary Fig. 2). RNA in situ hybridization showed that at early gastrula, *NvLsd1* is expressed throughout the embryo but more highly in scattered single cells in the ectoderm (Supplementary Fig. 1c). In late gastrula and planula stages, this pattern is maintained and strong expression is seen in the pharynx and endomesoderm (Supplementary Fig. 1d, e). By the polyp stage expression can be seen strongly in the tentacle region (Supplementary Fig. 1f). Since Lsd1 levels are subject to post-transcriptional regulation in other animals, we generated an endogenous allele tagged with eGFP and a FLAG tag using CRISPR-Cas9-mediated homologous recombination in order to better visualize NvLsd1 expression (Fig. 1b). We refer to the animals carrying this allele as *NvLsd1*^*GFP*^ animals. We confirmed the insertion of a single copy of GFP using PCR across the insertion site from genomic DNA (Supplementary Fig. 3a, d) and sequencing of this band. We also generated cDNA from *NvLsd1*^*GFP*^ animals and were able to clone a full-length cDNA containing the full *NvLsd1* coding sequence, the inserted sequences as well as an intact 3’ UTR (Supplementary Fig. 3b, c). In addition, we performed a western blot using an antibody against GFP and found a single band corresponding to the approximate size of the NvLsd1-GFP fusion protein (Supplementary Fig. 3e). Analysis of *NvLsd1*^*GFP*^ animals revealed that NvLsd1 is a ubiquitous protein found in all nuclei throughout development (Fig. 1c–l, Supplementary Fig. 4a) except on mitotic chromatin, which is devoid of NvLsd1-GFP signal (Supplementary Fig. 4b), similar to what was previously shown in mammalian cells^64^. As seen by RNA in situ hybridization, high levels of NvLsd1-GFP expression are seen in the pharynx and endomesoderm (Fig. 1f–h) and also in the tentacles later in development (Supplementary Fig. 4a). In addition, there are two populations of nuclei in the ectoderm; one with low and one with high levels of NvLsd1-GFP, from here on referred to as NvLsd1^low^ and NvLsd1^high^ cells (Fig. 1i–l). These two populations can also be distinguished by flow cytometry (Fig. 2f and Supplementary Fig. 5a). This heterogeneity in NvLsd1-GFP levels becomes more distinct as development progresses (Fig. 1i–l). Together this shows that NvLsd1 is ubiquitously expressed but that its cellular levels are regulated during development.

### NvLsd1 levels are high in differentiated neural cells

In order to determine the identity of the NvLsd1^low^ and NvLsd1^high^ cells we first performed EdU labeling experiments to mark proliferating cells. At late gastrula stage, we pulse labeled with EdU for 30 min or 4 h. 30 min incubation labels mostly S-phase cells while 4 h incubation leads to labeling of all cells in S-phase, G2 and likely early G1. This can be seen as after 4 h of EdU incubation all mitotic nuclei are EdU positive (Supplementary Fig. 4b). We performed this staining in *NvLsd1*^*GFP*^ animals and could see that in all cases NvLsd1^high^ cells were EdU negative (Fig. 2a and Supplementary Fig. 4b, c). This was also the case later at mid-planula stage (Supplementary Fig. 4d). Given the known role of Lsd1 in the mammalian nervous system we next investigated NvLsd1-GFP levels in different neural cell types. To do this we crossed *NvLsd1*^*GFP*^ animals to previously published neural reporter lines: *NvFoxQ2d*::mOrange which labels a population of sensory cells^53^, *NvElav1:*:mOrange which labels a non-overlapping population of sensory and ganglion cells^53,62^ and *NvNcol3:*:mOrange2 which labels cnidocytes^65^. In all cases, the mOrange^+^ cells have high levels of NvLsd1-GFP at late planula stage (Fig. 2b–d). In contrast, ectodermal gland cells labeled in the *NvPTx1*::mOrange2 line^66^ do not overlap with the NvLsd1^high^ population (Fig. 2e) at mid-planula stage, indicating that high levels of NvLsd1 are not a general feature of differentiated cells. For quantification, we compared the levels of NvLsd1-GFP in mOrange^+^ versus mOrange^-^ cells in each transgenic line (Supplementary Fig. 5a). This revealed that in the *NvElav1:*:mOrange, *NvFoxQ2d*::Orange, and *NvNcol3:*:mOrange2 lines, the mOrange^+^ cells fall largely within the NvLsd1^high^ population while in the *NvPTx1*::mOrange2 line far fewer mOrange^+^ cells are NvLsd1^high^ (Fig. 2f and Supplementary Fig. 5b). Together this shows that NvLsd1 levels are low in proliferating cells and are higher in differentiated neural cells.

### Loss of *NvLsd1* leads to developmental abnormalities

**Fig. 3:**
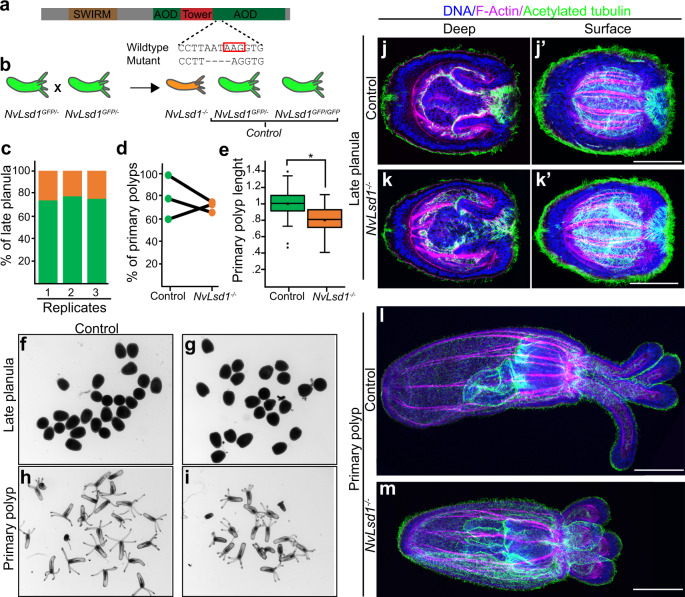
Generation and characterization of *NvLsd1* mutants. **a** Schematic showing the structure of the *NvLsd1* protein and the position and sequence of the mutation. The red box indicates the position of K644. **b** Cartoon depicting the crossing scheme used in all experiments with the *NvLsd1* mutants. **c** Quantification of the percentage of late planula which was control (in green) or *NvLsd1*^*−/−*^ (orange) across three independent biological replicates. **d** Percentage of control and *NvLsd1*^*−/−*^ late planula which developed into primary polyps. Three independent replicates are shown and matched samples are connected by a black line. **e** Box and whisker plot showing the measurement of body column length in control and *NvLsd1*^*−/−*^ primary polyps. The total body length is normalized to control. *n* = 50 (control), 51(*NvLsd1*^*−/−*^) animals examined over three independent experiments. Data shown are combined from all three replicates. Boxes indicate first and third quartile, median is represented by a line, mean is indicated by an asterix, whiskers indicate the minimum and maximum, and outliers are shown as dots. Statistical significance was determined using a two-tailed Student’s *t* test * *p* = 0.00000038344. Source data are provided as a Source Data file (**f**–**i**) Brightfield pictures of control or *NvLsd1*^*−/−*^ animals at late planula and primary polyp stage. Genotype is shown on top and stage is shown to the left. (**j**–**m**) Confocal images of immunofluorescence staining on control and *NvLsd1*^*−/−*^ late planula (**j**, **k**) and primary polyp (**l**, **m**). DNA is shown in blue, acetylated tubulin in green and F-actin in magenta. (**j**) and (**k**) show several projected confocal slices from the inside of the planula and j’ and k’ show several projected slices from closer to the surface. Images shown are representative samples from at least two independent replicates with >10 animals per replicate. Scale bar: 50 μm.

To analyze the function of *NvLsd1* we generated a mutant allele using CRISPR-Cas9. We targeted an exon containing a lysine at position 644 or K644 (K661 in humans) which is important for catalytic function and is located in a highly conserved portion of the protein (Supplementary Fig. 2). This residue interacts with the cofactor FAD and has been shown to be required for catalytic activity in vitro^8,10,11,67^, although mutation of it does not completely abolish enzymatic activity^68^. We generated a mutant line containing a 4 base pair deletion upstream of this lysine leading to a truncated protein missing a large portion of the amine oxidase domain (Fig. 3a). This mutation is similar to a zebrafish mutant that has been shown to be a null allele^69^. In order to trace the mutation we crossed mutants to *NvLsd1*^*GFP*^ animals generating animals with one GFP tagged allele and one mutant allele, *NvLsd1*^*GFP/-*^. In *NvLsd1*^*GFP/-*^ *x NvLsd1*^*GFP/-*^ crosses, 75% of the animals have at least one copy of the *NvLsd1*^*GFP*^ allele, hereafter called control, and the homozygous mutant animals are GFP negative, hereafter *NvLsd1*^*−/−*^ (Fig. 3b). This allowed us to distinguish *NvLsd1*^*−/−*^ animals from control by absence or presence of GFP signal, respectively. We confirmed the efficacy of this approach via sequencing (Supplementary Fig. 6a). *NvLsd1*^*−/−*^ animals develop normally until late planula (Fig. 3f, g). We counted the number of animals at late planula stage and show approximately Mendelian ratios of *NvLsd1*^*−/−*^ animals and controls, indicating no major defects in survival (Fig. 3c). We also quantified the percentage of animals that developed into primary polyps and saw no difference between mutants and controls (Fig. 3d). *NvLsd1*^*−/−*^ primary polyps are shorter than controls and have shorter tentacles but otherwise appear morphologically normal (Fig. 3e, h, i). A more detailed morphological examination using immunofluorescence also revealed no obvious differences at late planula stage (Fig. 3j, k). At primary polyp stage, although *NvLsd1*^*-/-*^ animals are shorter, all major morphological structures have formed, i.e. animals have four tentacles, a pharynx, and the internal tissue folds known as mesenteries (Fig. 3l, m). These animals fail to begin feeding normally but are capable of ingesting mashed artemia and therefore grow but much more slowly that their control counterparts and never develop fully (Supplementary Fig. 6b). The mutation, however, is not embryonic lethal and these animals can survive for greater than 3 months although they do not grow to maturity (Supplementary Fig. 6b). We can see from published RNAseq data^70^ that *NvLsd1* levels are high maternally (Supplementary Fig. 7a) and *NvLsd1*^*−/−*^ animals retain NvLsd1-GFP until mid-planula stage (Supplementary Fig. 7j–l). This NvLsd1-GFP protein must be derived from a maternal pool as these animals do not have an allele encoding NvLsd1-GFP. We therefore used an shRNA approach^71,72^ to reduce the maternal NvLsd1 pool. We can show by qPCR, that 4 h post-injection (prior to the major wave of zygotic genome activation^73^) this shRNA is capable of efficiently depleting the pool of maternal *NvLsd1* mRNA (Supplementary Fig. 7b). We injected this shRNA into embryos from crosses of either male or female heterozygous *NvLsd1*^*GFP*^ animals to wild types (Supplementary Fig. 7c–i). In animals with only zygotic *NvLsd1*^*GFP*^ (i.e. derived from male *NvLsd1*^*GFP*^ animals crossed to wild-type females) we see that shRNA injection efficiently removes NvLsd1-GFP (Supplementary Fig. 7d–f). In embryos derived from *NvLsd1*^*GFP*^ females, however, which also have maternally supplied *NvLsd1*^*GFP*^ mRNA/protein, the shRNA is not able to effectively remove the maternal pool (Supplementary Fig. 7g–i). We could therefore not use this approach to clarify the role of maternally supplied NvLsd1 protein. Together this shows that, although loss of zygotic *NvLsd1* is not lethal during embryonic stages, *NvLsd1*^*−/−*^ animals have altered development and do not survive until adulthood.

### Transcriptomics reveal a role for *NvLsd1* in cnidocytes

**Fig. 4:**
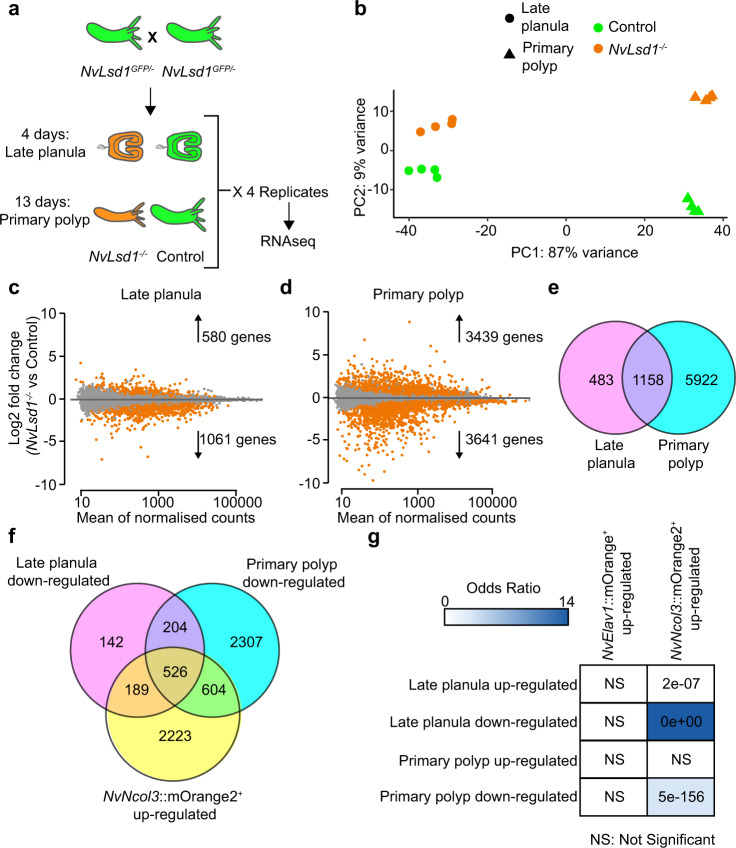
Transcriptomic analysis of *NvLsd1* mutants. **a** Cartoon depicting the experimental design for the transcriptome analysis of *NvLsd1* mutants. Controls are shown in green, and mutants are shown in orange. **b** PCA plot using the gene counts after variance stabilizing transformation (VST). Late planula samples are shown as circles and primary polyp as triangles. Experimental conditions are encoded with color: green for controls and orange for mutants. **c**, **d** MA-plots of up- and down- regulated genes between *NvLsd1*^*−/−*^ and control in late planula (**c**) and primary polyps (**d**). Differentially expressed genes are shown in orange at significance level of 0.05. **e** Venn diagram comparing all differentially expressed genes at late planula and primary polyp stages. **f** Venn diagram comparing genes downregulated in *NvLsd1*^*−/−*^ late planula and primary polyp with genes upregulated in *NvNcol3*::mOrange2^+^ cells. **g** Comparison of the overlap between up- and downregulated genes in *NvLsd1*^*−/−*^ late planula and primary polyps with those upregulated in *NvNcol3*::mOrange2^+^ and *NvElav1*::mOrange^+^ cells using the GeneOverlap R package. The strength of the blue color indicates the odds ratio and numbers indicate the *p*-value, calculated using one-sided Fisher’s exact test.

Next, we analyzed the transcriptional changes occurring due to mutation of *NvLsd1*. We performed RNAseq on *NvLsd1* mutants and controls (consisting of *NvLsd1*^*GFP/-*^ and *NvLsd1*^*GFP/GFP*^ *animals*) in quadruplicate at two different developmental stages: four-day-old late planula and 13-day-old primary polyp (Fig. 4a and Supplementary Data 3). Principal component analysis (PCA) showed that, as expected, the majority of variance separates samples based on developmental stage (the first principal component, PC1). However, the second principal component (PC2) separates animals based on whether they are *NvLsd1*^*−/−*^ or control (Fig. 4b). Differential gene expression analysis revealed 1641 differentially expressed genes in late planula (580 upregulated and 1061 downregulated) and 7080 in primary polyp (3439 upregulated and 3641 downregulated) (Fig. 4c, d). The lower number of genes differentially expressed at late planula stage correlates with the less severe phenotype seen at this stage (see also later sections) but it is important to note that some maternal *NvLsd1* persists late in development and could be masking the effects of zygotic loss of *NvLsd1* (Supplementary Fig. 7j–l). Comparing genes differentially expressed at the different stages revealed that 1158 out of 1641 genes (**≈**71%) differentially expressed in late planula are also differentially expressed in primary polyps (Fig. 4e). *NvLsd1* itself was among the downregulated genes in both primary polyp and late planula samples potentially due to some level of autoregulation or nonsense-mediated decay (Supplementary Fig. 8a). We also checked for expression of all annotated histone demethylases in *Nematostella* but did not find any of them upregulated in *NvLsd1* mutants. Given the high levels of NvLsd1 in differentiated neural cells, we compared the differentially expressed genes to a previously published transcriptome from *NvElav1*::mOrange^+^ neurons^52^ and to the transcriptome of *NvNcol3*::mOrange2^+^ cnidocytes (Supplementary Data 4) also produced here. Importantly, both these transcriptomes were generated at the same stage as the transcriptome of the *NvLsd1* mutant polyps (13-day primary polyp). We observed only limited overlap of genes upregulated in mutants with genes upregulated in either cell population (~16–23%) (Supplementary Fig. 8b, c). However, when we looked at genes downregulated in *NvLsd1* mutants, we saw that for both late planula and primary polyp there is a larger overlap with genes upregulated in cnidocytes (~67% and ~31%, respectively) (Fig. 4f) but not with genes upregulated in neurons (~12% and ~13% respectively) (Supplementary Fig. 8d). These include genes known to be expressed in cnidocytes such as NEP6 and NEP16^74^ (Supplementary Fig. 8a).To test if these overlaps were statistically significant, we used the GeneOverlap R package (Shen and Sinai, 2020) which implements Fisher’s exact test to calculate if the overlap is significant and provides several metrics, such as *p*-value (significance) and Odds ratio (strength of association). An Odds ratio of 1 or less indicates no association while increasing Odds ratios above 1 indicate increasing association between the gene sets. The overlap between genes downregulated in *NvLsd1* mutants and *NvNcol3*::mOrange2^+^ cnidocytes was significant while the overlap with genes upregulated in *NvElav1*::mOrange^+^ neurons was not (Fig. 4g). The overlap between genes upregulated in *NvLsd1* mutants and those upregulated in *NvElav1*::mOrange^+^ was also not significant. The genes upregulated in the primary polyp stage did not overlap significantly with those upregulated in *NvNcol3*::mOrange2^+^ cnidocytes, however, those upregulated in late planula did have a significant overlap albeit with a relatively low Odds ratio and higher p-value (Fig. 4g). We also performed more stringent analysis for the selection of differentially expressed genes by raising the log2 fold change threshold from 0 to 1, and hence, removing genes with less substantial changes due to mutation of *NvLsd1* (standard DESeq2 approach). We found the same results although we now see an overlap between genes upregulated in primary polyp and *NvNcol3*::mOrange2^+^ cells although with a very high p-value and low Odds ratio (Supplementary Fig. 8e). To complement this analysis, we compared genes downregulated in *NvLsd1* mutants to the *Nematostella* single-cell atlas^61^ in order to ask whether the markers of any specific cell populations were enriched among those affected by loss of *NvLsd1*. In comparing the late planula/larval data sets we see two metacells (representing putative cell types/states^75^) that stand out and which correspond to the cnidocytes (Supplementary Fig. 9b). In the primary polyps there is a clear enrichment of downregulated genes in the cnidocyte metacells (Supplementary Fig. 9a), confirming our analyses of the *NvNcol3*::mOrange2^+^ transcriptome. The same comparison using genes upregulated in *NvLsd1* mutants showed no such enrichment. A more detailed look at the cnidocyte dataset revealed that in fact the downregulated genes were enriched in more differentiated cnidocytes (both nematocytes and spirocytes) rather than in the less differentiated cells (Supplementary Fig. 9c, d). Together this indicates that loss of *NvLsd1* affects cnidocyte formation.

### Loss of *NvLsd1* causes defects in cnidocyte differentiation

**Fig. 5:**
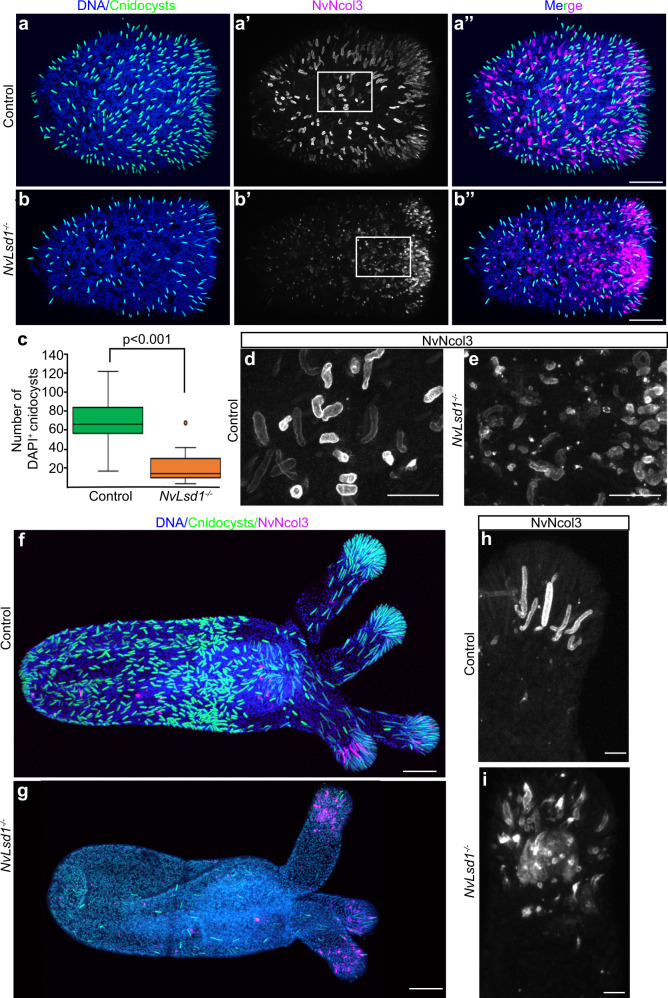
*NvLsd1* mutants have defects in cnidocyte differentiation. **a**, **b** Confocal images of immunofluorescence staining on control and *NvLsd1*^*−/−*^ late planula showing DNA in blue, mature cnidocysts in green and NvNcol3 in magenta. **c** Box and whisker plot showing quantification of mature DAPI^+^ cnidocysts from control and *NvLsd1*^*−/−*^ late planula. The number of cnidocysts in a 100 μm × 100 μm area in the center of each larva was counted. Boxes indicate first and third quartile, median is represented by a line, whiskers indicate the minimum and maximum, and outliers are shown as dots. Statistical significance was calculated using a two-tailed Student’s *t* test, *p* = 0.00041. *n* = 9 animals per condition. The experiment was conducted with two independent replicates, which yielded the same result. Data shown are from a single experiment. Source data are provided as a Source Data file. **d**, **e** Close ups of the regions highlighted in a’ (**d**) and b’ (**e**) showing NvNcol3 staining in gray. **f**, **g** Confocal images of immunofluorescence staining on control and *NvLsd1*^*−/−*^ primary polyps. DNA is shown in blue, mature cnidocysts in green and NvNcol3 in magenta. **h**, **i** High magnification of NvNcol3 staining in the tentacles of primary polyps in f (**h**) and g (**i**) showing NvNcol3 staining in gray. Images shown are representative samples from at least two independent replicates with >10 animals per replicate. Scale bars: 50 μm (**a**, **b**, **f**, and **g**), 20 μm (**d**, **e**) and 10 μm (**h**, **i**).

**Fig. 6:**
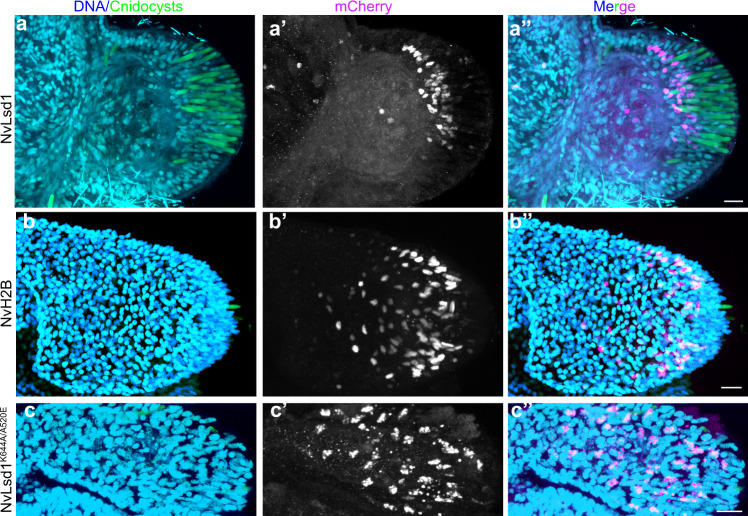
NvLsd1 is required cell autonomously for cnidocyte differentiation. **a**–**c** Immunostaining on *NvLsd1*^*−/−*^ animals injected with *NvPOU4*:*NvLsd1-mCherry* *(n = 9)* (**a**), *NvPOU4*:*NvH2B-mCherry (n = 16)* (**b**) or *NvPOU4*:*NvLsd1*^*K644A/A520E*^*-mCherry (n = 6)* (**c**). DNA is shown in blue, DAPI^+^ cnidocysts in green and mCherry in magenta. Experiments were performed three times independently and the total number of animals analysed for each condition are indicated (n). Scale bars: 10 μm.

Having found a significant overlap between downregulated genes in the *NvLsd1*^*−/−*^ animals and those upregulated in cnidocytes, we next looked at cnidocyte differentiation in *NvLsd1*^*−/−*^ animals. Cnidocytes are cnidarian-specific neural cells that are responsible for prey capture and defense. Cnidocytes contain a specific organelle, called the cnidocyst, which forms from a large post-Golgi compartment. This pressurized organelle contains a tubule that is extruded like a harpoon upon explosive rupture of the cnidocyst^76^. Different stages in the development of this neural cell type can be visualized by monitoring the formation and maturation of the cnidocyst. An antibody against NvNcol3 labels the forming cnidocyst wall but does not label the fully mature cnidocyst^77^. A modified DAPI staining protocol, on the other hand, labels the matrix of the mature cnidocyst^78^. Staining at the late planula stage revealed an overall reduction in the number of mature cnidocysts in *NvLsd1*^*−/−*^ animals (Fig. 5a–c). NvNcol3 staining in controls shows developing cnidocysts with a regular, elongated shape (Fig. 5a, d). In *NvLsd1*^*−/−*^ animals, however, patchy and irregular staining was observed suggesting that although cnidocytes are present, they are not able to complete their differentiation (Fig. 5b, e). At polyp stage there is an even larger reduction in mature cnidocysts (Fig. 5f, g) and the same change in the pattern of NcNcol3 staining is observed (Fig. 5f–i). Since *NvLsd1* is ubiquitously expressed, we tested whether the role in cnidocyte development was cell autonomous by a cell-type-specific rescue approach. We expressed *NvLsd1* under the control of the *NvPOU4* promoter that, in the tentacles, is primarily expressed in post-mitotic cnidocytes^52^. In the background of *NvLsd1* mutants, we generated F0 mosaic transgenics expressing either *NvPOU4::NvLsd1* or *NvPOU4*::*NvHistoneH2B* (as a control) and stained for cnidocytes. In some cases, we saw small patches of expression with few positive nuclei. We exclude these from our analysis as, in uninjected *NvLsd1* mutants, there is always a small number of cnidocytes in the tentacles (Fig. 5g) making it difficult to analyze the effects of transgene expression in just a few cells. In larger patches, expression of *NvPOU4*::*NvLsd1* always led to a rescue of the cnidocyte phenotype as seen by corresponding, overlapping patches of DAPI^+^ cnidocysts (*n* = 9/9) (Fig. 6a). *NvPOU4*::*NvHistoneH2B* expression, however, never led to such an effect (*n* = 16/16) (Fig. 6b). We also generated a mutant version of *NvLsd1* bearing two single amino acid changes that completely block catalytic activity in human LSD1^68^, NvLsd1^A520E/K644A^. The expression of this mutant was unable to rescue the effect of loss of *NvLsd1* in cnidocytes (*n* = 6/6) (Fig. 6c). Together this data shows an important role for *NvLsd1* in cnidocyte maturation.

Although there was no significant overlap between genes differentially expressed in *NvLsd1*^*−/−*^ animals and genes upregulated in *NvElav1*::mOrange^+^ neurons, NvLsd1 levels were shown to also be high in these cells. We therefore analyzed whether loss of *NvLsd1* affects the *NvElav1*::mOrange^+^ nervous system. In mutant primary polyps, the expression of the transgenic reporter is less uniform, with many mOrange^+^ punctae, which are not present in controls, and with an apparent expansion of fluorescence into endodermal epithelial cells (Supplementary Fig. 10a, b). We could not, however, identify an effect on the overall architecture of the *NvElav1*::mOrange^+^ nervous system or specific changes to the morphology of individual neurons. We also looked at *NvFoxQ2d*::mOrange^+^ neurons but did not see gross changes in these cells in *NvLsd1*^*−/−*^ animals compared with controls (Supplementary Fig. 10c, d). Additionally, neither *NvElav1* nor *NvFoxQ2d* are differentially expressed in *NvLsd1* mutants.

## Discussion

Lsd1 is an important chromatin regulator involved in several steps of neurogenesis in mammals. Here we show that Lsd1, in the sea anemone *Nematostella*, is developmentally regulated and plays a crucial role in the differentiation of cnidocytes, a cnidarian-specific neural cell type.

The levels of chromatin regulators are often tightly controlled in a cell and tissue-specific manner in bilaterians. Lsd1 is a prime example of this with many examples of its complex regulation. In *Nematostella*, we have shown that NvLsd1 levels are also regulated during neurogenesis with higher levels seen in differentiated cells. This is similar to observations in human cells^18,35^. While we did not observe high levels of NvLsd1 in *NvPTx1*::mOrange2-expressing secretory cells, we cannot exclude that NvLsd1 levels are high in other non-neural cell types which we have not analyzed. The regulation of NvLsd1 appears to be both transcriptional and post-transcriptional. We see by RNA in situ hybridization that *NvLsd1* mRNA levels are heterogeneous (Supplementary Fig. 1c–f) and it is also upregulated in the *NvElav1*::mOrange^+^ cells indicating regulation at the transcriptional level. We have also shown that some maternal NvLsd1 lasts late into development and we have shown that, although levels are much lower overall, there is still a heterogeneity between cells in the amount of maternal protein present at late planula stage (Supplementary Fig. 7j–l). As this heterogeneity is derived from maternal mRNA and/or maternal NvLsd1 protein, it must represent a level of post-transcriptional control of NvLsd1 levels. It will be interesting in the future to dissect the transcriptional and post-transcriptional regulation of Lsd1 and compare it to that of mammals to understand if there are conserved processes involved.

In mice, Lsd1 mutation is lethal at early embryonic stages^15^ while in zebrafish, *D. melanogaster* and *C.elegans*, mutants are viable but display several phenotypic abnormalities^36,37,69^. Here we show that loss of zygotic *NvLsd1* is not embryonic lethal in *Nematostella* and in fact, animals survive for many months. It is, however, possible that an essential role for *NvLsd1* in early development is masked by maternally provided *NvLsd1* present in the zygotic *NvLsd1* mutants. Indeed, in mice maternal Lsd1 is required for the maternal to zygotic transition^79,80^ and in fish it has also been proposed that the large maternal pool of Lsd1 may be the reason for a lack of more severe phenotypes early in development^69^. We have not been able to grow *NvLsd1*^*−/−*^ animals to sexual maturity likely due to their inability to feed/grow properly, and although we were able to utilize shRNAs to effectively deplete maternal mRNAs this did not completely remove all maternally supplied NvLsd1. Therefore, studying this maternal pool of *NvLsd1* will require the establishment of new tools in *Nematostella*.

The function of Lsd1 in the nervous system has so far only been well demonstrated in mammals where Lsd1 is playing many different roles. Here we show that *NvLsd1* also has a role in the nervous system of *Nematostella*. The strongest evidence for this is the requirement for *NvLsd1* for proper cnidocyte differentiation. Cnidocytes, although a cnidarian-specific cell type, are bona fide neural cells^48,49^. Loss of *NvLsd1* leads to a loss of mature cells but does not appear to lead to major defects in cell specification. This can be seen by the continued presence of NvNcol3^+^ immature cnidocytes despite the severe reduction in mature cells. *NvNcol3* is also not differentially expressed in mutants. By comparison to the *Nematostella* single-cell atlas^61^, we see that genes downregulated in the *NvLsd1* mutants are enriched in more differentiated cnidocytes (including both spirocytes and nematocytes) and not in the less differentiated cells. Additionally, transcription factors *NvPaxA* and *NvPOU4*, which are known regulators of cnidocyte differentiation^52,58^, are not differentially expressed in either late planula or primary polyps. In contrast, *Cnido-Jun* and *Cnido-Fos1* are downregulated in mutants at both stages (Supplementary Fig. 8a). These two transcription factors have recently also been shown to be required for cnidocyte differentiation^65^. This indicates that the effect of loss of *NvLsd1* occurs downstream of *NvPaxA* and *NvPOU4* but upstream of other regulators like *Cnido-Jun* and *Cnido-Fos1*. We do not, however, have a full picture of the gene regulatory network involved in cnidocyte differentiation and it is therefore currently difficult to place *NvLsd1* function within this network. The rescue experiments further showed that expressing *NvLsd1* in post-mitotic cnidocytes is sufficient for cnidocyte development, revealing that *NvLsd1* has a cell autonomous role and is not required for the initial specification of this cell type. This combined paradigm of an increase of Lsd1 levels as differentiation progresses and a requirement during late stages is also seen in certain cell types in vertebrates such as rod photoreceptors^32^. The fact that the NvLsd1^K644A/A520E^ mutant is not capable of rescuing the cnidocyte phenotype suggests that catalytic function is required for the role of NvLsd1 in cnidocytes. We cannot exclude, however, that this mutant form also affects protein-protein interactions of NvLsd1, as has been shown for other Lsd1 mutations^81^. Histone demethylase activity of Lsd1 proteins is conserved in unicellular and multicellular eukaryotes^7,36,82^, but it remains possible that NvLsd1 has relevant non-histone target proteins, as has been shown to be the case in other contexts^83^. A rigorous examination of NvLsd1 interaction partners/targets would aid in this greatly.

The level of NvLsd1 is also higher in *NvElav1:*:mOrange^+^ and *NvFoxQ2d:*:mOrange^+^ neurons. We were, however, unable to identify a morphological phenotype in *NvFoxQ2d:*:mOrange^+^ cells upon mutation of *NvLsd1*. We also see only a mild effect on the *NvElav1:*:mOrange^+^ nervous system. While this is in line with the small number of genes differentially expressed in mutants and upregulated in *NvElav1*::mOrange^+^ cells, we cannot rule out that further morphological or functional alterations will become detectable with more sophisticated experimental tools. Although we have focused here on neural cells it is possible that loss of NvLsd1 has effects on other cell populations. The development of more tools to study non-neural cells will facilitate the analysis of *NvLsd1* function in other cell lineages in the future.

In conclusion, we have shown that NvLsd1, a ubiquitously expressed chromatin modifier, is developmentally regulated in *Nematostella* and that it is required cell autonomously for the differentiation of cnidocytes. These observations allow us to speculate that developmental regulation of chromatin modifiers and their integration into cell differentiation programs arose early in animal evolution and thus constitute an ancient feature of metazoan development.

## Methods

### Animal care and maintenance

Polyps of the *Nematostella* laboratory strain CH2 x CH6^84^ were maintained at 18–19 °C in 1/3 filtered sea water [*Nematostella* medium (NM)], and male and female adult animals of unknown age were spawned as described previously^85^. Fertilized eggs were removed from their jelly packages by incubating in 3% cysteine in NM for 20 min followed by extensive washing in NM. Embryos were reared at 21 °C and were fixed at 4 (cleavage stage), 8 (early blastula), 12 (mid blastula), 20 (early gastrula), 30 (late gastrula), 48 (early planula), 72 (mid-planula), 96 (late planula), 120 (tentacle bud) hours post-fertilization or at 13 days (primary polyp). The sex of embryos was not determined. Animal maintenance and experimentation were conducted in compliance with the animal welfare regulations at the University of Bergen.

#### Immunofluorescence (IF)

Animals older than 72 h were anesthetized with MgCl_2_ and then killed quickly by adding a small volume (20–30 μl/ml) of 37% formaldehyde directly into the media. They were then fixed in ice cold 3.7% formaldehyde in PBTx [PBS(Phosphate Buffered Saline) + 0.2% Triton X-100] for 30–60 min (when staining for NvLsd1-GFP short fixations yield better staining) or for >60 min or overnight (o/n) (for all other antibodies) at 4 °C. Samples were washed >4 times in PBTx at RT, blocked in Block (3% BSA / 5% Goat serum in PBTx) for > 1 h at RT and incubated in primary antibody diluted in Block o/n at 4 °C. Samples were then washed extensively in PBTx (>5 washes for 2 h or more) at RT, blocked for 1 h at RT in Block and incubated o/n or over the weekend in secondary antibody diluted in Block at 4 °C. If Phalloidin staining was performed, Alexa Fluor™ 488 or 633 Phalloidin (Thermo Fisher Scientific, A12379/ A22284) was added here at 1:50-1:100. Samples were then incubated in Hoechst 33342 (Thermo Fisher Scientific, 62249) at 1:100 in PBTx for 1 h at RT followed by extensive washing in PBTx (>5 washes for 2 h or more). Animals were mounted in ProLong™ Gold Antifade Mountant with DAPI (Thermo Fisher Scientific, P36935) and imaged on a Leica SP5 confocal microscope. Antibodies and dilutions are listed in Supplementary Table 1.

#### DAPI staining for cnidocysts and counting

DAPI staining for cnidocysts was performed as previously published^60,78^ with slight modifications. Animals were processed as for IF with the addition of 10 mM EDTA to all solutions. Following the final PBTx wash, the samples were washed twice with MilliQ H_2_O and then incubated in 200 μg/ml DAPI in milliQ H_2_O o/n at RT. The samples were then washed once with MilliQ H_2_O, twice with PBTx with 10 mM EDTA and mounted and imaged as for IF.

For counting of cnidocysts, late planula was imaged on a Leica SP5 confocal microscope and a maximum intensity projection of one half on the planula was generated using Fiji^86^. The number of DAPI^+^ cnidocysts was then counted in a 100 μM square located in the middle of the planula. At least 10 planula were used per condition/replicate. The experiment was repeated twice and the data shown are the results of one replicate. Statistical significance was assessed using a Student’s *t* test (two-tailed, equal variance).

#### EdU labeling

Animals used in Edu labeling experiments were incubated in 10 mM EdU in NM for the desired time and then treated for IF as described. After the final set of PBTx washes EdU incorporation was visualized using the Click-iT™ EdU Imaging Kit with Alexa Fluor™ 488 or 647 (Thermo Fisher Scientific, C10337/C10337) following the manufacturer’s protocol. Samples were mounted and imaged as for IF.

#### Flow cytometry and FACS

Flow cytometry and FACS sorting were performed as previously described^87^. Briefly, animals were dissociated in 0.25% Trypsin (Gibco, 27250018) in Ca- Mg-free *Nematostella* medium (CMFNM) (154 mM NaCl, 3.6 mM KCl, 2.4 mM Na_2_SO_4_, 0.7 mM NaHCO_3_) supplemented with 6.6 mM EDTA, pH 7.6–7.8. Cells were centrifuged at 800 *g* for 10 min, resuspended in ice cold 0.5% BSA in CMFNM (pH 7.6–7.8), filtered through a 40 μM filter and stained with Hoechst 33342 (Thermo Fisher Scientific, 62249) at a 60 μg/ml at RT for 30 min. Samples were then diluted 1:1 with ice cold 0.5% BSA/CMFNM and stained with 60 μl/ml 7-AAD (BD, 559925) for >20 min on ice. Flow cytometry was performed on a BD Fortessa and sorting was carried out on a BD FACSAria II with a 100 μm nozzle. Data were analyzed using FlowJo v10.7.

#### RNA extraction, library preparation and sequencing

For the RNAseq of *NvNcol3*::mOrange2^+^ cells, RNA extraction was performed as previously published^87^. Cells were sorted directly into 0.5% BSA/ CMFNM at 4 °C and centrifuged at 800 g for 10 min at 4 °C. Most of the liquid was removed and 3 volumes of TRIzol LS reagent (Invitrogen, 10296028) was added. Samples were vortexed extensively and incubated at RT for 5 min before being flash frozen and stored at −80 °C. The samples were processed using Direct-zol RNA MicroPrep columns (Zymo Research, R2060) including on column DNase digestion. cDNA was prepared from 400 pg of total RNA using the Smart-Seq 2 method with 16 pre-amplification PCR cycles^88^. NGS libraries were prepared using the home-made tagmentation-based method^89^. Briefly, 125 ng of cDNA was tagmented using home-made Tn5 loaded with annealed linker oligonucleotides for 3 min at 55 °C. Reactions were inactivated by adding 1.25 ml of 0.2% SDS and incubation for 5 min at RT. Indexing and amplification were done using the KAPA HiFi HotStart PCR kit (Sigma-Aldrich, KK1508) with Index oligonucleotides (sequences were adapted from Illumina).

For RNAseq on late planula and primary polyp, 50 animals per sample were lysed in 500 μl TRIzol reagent (Invitrogen, 15596026) by vortexing extensively and incubated at RT for 5 min. 100 μl of chloroform was added and mixed vigorously and the aqueous component was isolated using MaXtract High Density tubes (Qiagen, 129046) using the manufacturer’s protocol. One volume of 100% Ethanol was added to the aqueous phase. This was then processed using an RNeasy Micro Kit (Qiagen, 74004) using the manufacturers protocol including on column DNase digestion using the RNase-Free DNase Set (Qiagen, 79254). Libraries were prepared using the NEBNext Ultra II Directional RNA Library Prep Kit for Illumina (E7760L), with the following changes: 25 ng RNA input, 1/100 adaptor dilution, 14 PCR cycles. Libraries were sequenced using a 75 bp single end sequencing on a NextSeq500 machine (Illumina).

RNA quality was assessed using an RNA 6000 Pico Kit (Aligent, 5067-1513) and concentration determined using the Qubit™ RNA HS assay kit (Invitrogen, Q32852). RNAseq data are deposited at ArrayExpress (https://www.ebi.ac.uk/arrayexpress) with accession numbers MTAB-9556 (*NvNcol3*::mOrange2) and E-MTAB-9562 (*NvLsd1* mutants), respectively.

#### sgRNA synthesis

For the generation of the *NvLsd1* mutant allele the sgRNA was produced using a template generated by primer annealing. A PCR was set up containing 5 μl of each primer (100 mM), 2 μl dNTPs (10 mM each), 2 μl Q5 polymerase (NEB, M0491), 10 μl Q5 reaction buffer and 31 μl H_2_O with the following protocol: 98 °C, 90 s; 55 °C, 30 s; 72 °C, 60 s. This was purified using a PCR clean up kit (Promega, A9281). The sgRNAs were synthesized using the MEGAscript™ T7 Transcription Kit (Invitrogen, AMB13345) including the DNase treatment.

For the generation of the *NvLsd1*^*GFP*^ line, sgRNAs were produced using the EnGen^®^ sgRNA Synthesis Kit (NEB, E3322S). Primers are given in Supplementary Table 2.

In both cases, sgRNAs were precipitated by adding 1:1 LiCl (7.5 M) (Invitrogen AM9480) and incubating at −20 °C for 30 min followed by centrifugation at full speed at 4 °C for 15 min and extensive EtOH washes. The concentration was calculated using a Nanodrop.

#### CRISPR-Cas9 injections and genotyping

*NvLsd1* mutants were produced similarly to previously published^90,91^. Eggs were injected with a mix containing sgRNA (130 ng/μl), Cas9 (PNA Bio, CP01) (500 ng/μl) and 50 ng/μl Dextran, Alexa Fluor™ 568 (Invitrogen, D22912) (200 ng/μl in 1.1 M KCl) that was incubated at 37 °C for 5–10 min prior to injection. Injected animals were raised to sexual maturity and crossed to wild types. F1 offspring were analyzed by sequencing in order to identify an F0 carrying the desired mutation. Individual F1s were placed in tubes, the NM removed and 100% EtOH added. After 5 min this was removed and the tubes were placed at 50 °C for 45 min to allow the remaining EtOH to evaporate. 50 μl genomic extraction buffer (10 mM Tris pH8, 1 mM EDTA, 25 mM NaCl, 200 μg/μl ProteinaseK) was added to each and incubated at 50 °C for 2 h and 98 °C for 15 min. 2 μl of this was used for PCR and sequencing. Once an F0 carrier was identified, the remaining F1 offspring from that carrier were genotyped using a piece of tissue in order to generate a pool of F1 heterozygous animals.

For generating the *NvLsd1*^*GFP*^ line, the repair template was produced by PCR using primers given in Supplementary Table 2 and an in-house eGFP plasmid as template. The PCR product was gel extracted (Promega, A9281). Wild-type embryos were injected with a mix containing two sgRNAs (56.25 ng/μl each), repair template (25 ng/μl), Cas9 (PNA Bio, CP01) (500 ng/μl) and 50 ng/μl Dextran, Alexa Fluor™ 568 (Invitrogen, D22912) (200 ng/μl in 1.1 M KCl) that was incubated at 37 °C for 5–10 min prior to injection. Animals were screened for GFP fluorescence in the days following injection and GFP^+^ animals were grown to maturity and crossed to wild types in order to identify a carrier. A single F1 male was used to generate the line and all animals used in further analysis are offspring of this animal. The insertion was validated using PCR and western blotting (See Supplementary Fig. 2).

#### PCR, cloning, and sequencing

For generating cDNA for PCR, RNA was extracted for the RNAseq of mutants. The SuperScript™ III first-strand synthesis system (Invitrogen, 18080051) was used to generate cDNA. The gDNA for PCR was extracted for genotyping except several pooled animals.

All PCRs were performed with Q5 polymerase and primers are listed in Supplementary Table 2.

For cloning of the *NvLsd1-GFP* cDNA and genomic DNA fragments (Supplementary Fig. 2) the fragments were cloned using the CloneJET PCR Cloning Kit (Thermo Fisher Scientific, K1231). For the cloning of the *NvLsd1* cDNA primers were designed using the gene model (Nve23413, JGI: v1g105193) and the amplified fragment was cloned into a pCR4 backbone using the NEBuilder® HiFi DNA Assembly master mix (NEB, E2621). For sub-cloning into the *NvPOU4*::mCherry plasmid^52^ both the backbone and the insert were amplified by PCR and assembled using the NEBuilder^®^ HiFi master mix. The *NvH2B* was amplified from primers designed against gene model Nve18479 (Genebank: XM_001635370.2). To generate the NvLsd1^K644A/A520E^ mutations we used synthesized fragments of *NvLsd1* including the mutations. We then amplified the *NvPOU4*::NvLsd1-mCherry plasmid to remove the corresponding part of *NvLsd1* and inserted the fragment using the NEBuilder^®^ HiFi master mix.

Plasmids were sequenced using either BigDye™ Terminator v3.1 sequencing kit (Applied Biosystems, 4337458) and analyzed in house or samples were sequenced commercially using the GENEWIZ sanger sequencing service.

#### Bioinformatic analysis

The quality of raw RNA-seq reads was initially assessed with FastQC software v.0.11.8^92^ and filtering was performed with fastp v.0.20.0^93^ in default settings. Reads which passed quality control were mapped with STAR aligner v.2.7.3a^94^ in default settings to *N.vectensis* genome (https://mycocosm.jgi.doe.gov/Nemve1/Nemve1. home.html)^95^ using NVE gene models (https://figshare.com/articles/Nematostella_vectensis_transcriptome_and_gene_models_v2_0/807696). Downstream analysis was performed using R v.4.0.2^96^ and Bioconductor packages (https://www.bioconductor.org). Briefly, aligned reads were counted with ‘summarizeOverlaps‘ function from the package ‘Genomic Alignments‘ v.1.24.0^97^ and genes with less than 10 counts in at least 4 biological replicates for one condition were filtered out. Differential expression analysis was performed using ‘DESeq2‘ package v.1.28.1^98^. Overlap between up- and/or down- regulated genes across different conditions was assessed with ‘GeneOverlaps‘ package v.1.23.0^99^.

#### Analysis of Lsd1 mutant survival, metamorphosis, and growth

In order to assess the survival of *NvLsd1* mutant embryos Lsd1^GFP/-^ animals were in-crossed. At 48 h, ~100 embryos were sorted as having “normal” morphology independent of whether they were GFP^+^. At 96 h these animals were genotyped based on whether they were GFP^+^ (control) or GFP^-^ (mutant). The same animals were separated into 2 dishes based on their genotype and grown to primary polyp. The number of the animals that developed properly was then counted at this stage and is shown as a percentage of the number of animals present at 4 days. These were then imaged on a Nikon SMZ18 with a Nikon DS-Qi2 camera, using the same magnification and settings and the length of each animal was determined using the measure tool in Fiji^86^. Length was normalized by dividing by the mean length of the control animals. Statistical significance was assessed using the Student’s *t* test (two-tailed, equal variance). Levene’s test was used to determine equal variance and normality was tested using the Kolmogorov–Smirnov test.

#### Transgenesis

In order to generate F0 mosaic transgenics we used I-Sce1 mediated transgenesis as previously described^100^ with minor modifications. Eggs were injected with a mix containing: plasmid DNA (10 ng/ul), ISce1 (1U/ul) (NEB, R0694), Dextran Alexa Fluor™ 568 (100 ng/ul), CutSmart buffer (1x). The mix was incubated for 30 min at 37 °C before injection.

### Reporting summary

Further information on research design is available in the Nature Research Reporting Summary linked to this article.

## Supplementary information

Supplementary Information

reporting summary

Description of Additional Supplementary Files

Supplementary Data 1

Supplementary Data 2

Supplementary Data 3

Supplementary Data 4

## Data Availability

The RNAseq data generated in this study have been deposited in Array Express under accession codes E-MTAB-9556; *NvNcol3*::mOrange3 transcriptome data) and E-MTAB-9562; *NvLsd1* mutant transcriptome data). The *NvElav1*::mOrange transcriptome data is previously published^52^ and is available at Array Express with accession number E-MTAB-8794. Source data for graphs in Figs. 3e and 5e are provided as a Source Data file. Source data are provided with this paper. The RNAseq data can be explored via a Shiny app (https://nmve.shinyapps.io/shiny/).

## Source data

Source Data

## Code availability

Custom R script used for analysis of single cell data (See supplementary information) is available on github (https://github.com/IanKouzel/nve_lsd1).
