# Supplementary material for: Histone demethylase Lsd1 is required for the differentiation of neural cells in Nematostella vectensis
Source: Nat Commun. 2022 Jan 24;13:465. doi: 10.1038/s41467-022-28107-z (PMC8786827; doi:10.1038/s41467-022-28107-z)
Supplement: Supplementary file 1 — Supplementary Information [file 41467_2022_28107_MOESM1_ESM.pdf]

Supplementary Information for:

Histone demethylase Lsd1 is required for the differentiation of neural cells in the cnidarian *Nematostella vectensis*

James M Gahan[1], Ian U. Kouzel[1], Kamilla Ormevik Jansen[1], Pawel Burkhardt[1], Fabian Rentzsch[1,2]

Corresponding authors: James M Gahan, Fabian Rentzsch

Emails: james.gahan@uib.no, fabian.rentzsch@uib.no

This PDF includes:

Supplementary Methods

Supplementary Tables 1 and 2

Supplementary Fig. 1-10

Supplementary references

Supplementary Methods

Phylogenetic analysis. Phylogenetic analysis was performed on AOD domains of Lsd/KDM1 proteins. Amino acid sequences of AOD domains were retrieved from complete KDM1 sequences based on coordinates in Pfam data base [1] and are provided as supplementary data file 1. Multiple sequence alignment of AOD domains was created using MAFFT program v7.475 [2] in default settings (Supplementary Data 2). The maximum-likelihood tree was inferred using IQ-TREE software v 1.6.12 [3] using default settings and visualized using iTOL (https://itol.embl.de). Consensus tree was constructed from 1000 bootstrap trees.

RNA *In-situ* hybridization. Animals were fixed in ice cold 0.2% glutaraldehyde/3.7% formaldehyde in NM for 1.5 minutes followed by 1 hour at 4°C in 3.7% formaldehyde in PBT (PBS + 0.1% Tween 20). Animals were washed several times in PBT at room temperature (RT) and dehydrated through a series of methanol washes and stored in 100% methanol at -20°C. Probes were generated with T3 RNA polymerase (Roche, 11031163001) and DIG RNA labelling mix (Roche 11277073910). *In situ* hybridization was performed as previously described [4, 5]. Samples were imaged on a Nikon Eclipse E800 compound microscope with a Nikon Digital Sight DSU3 camera.

Western blotting. Protein extraction was performed on Lsd^{GFP} or wild-type late planula. Animals were placed in RIPA buffer (150mM NaCl, 50mM Tris pH8, 1% NP40, 0.5% DOC, 0.1% SDS) supplemented with cOmplete EDTA-free Protease Inhibitor Cocktail (Roche, 4693159001) and homogenized by passing through a 27G needle. Samples were incubated on ice for 30 minutes and mixed by passing through the needle every 5 minutes and centrifuged at full speed for 15 minutes @ 4°C. The

supernatant was kept and the protein concentration quantified using the Qubit™ Protein Assay (Invitrogen, Q33212). 30 µg of protein was used per lane, mixed 1:1 with 2X Laemmli sample buffer (0.1M TrisHCl pH 6.8, 2% SDS, 20% Glycerol, 4% β-mercaptoethanol 0.02% Bromophenol blue) and boiled for 5 minutes before loading. PageRuler™ Plus prestained protein ladder, 10 to 250 kDa (Thermo Scientific, 26619) was used. SDS PAGE was performed using 7.5% Mini-PROTEAN® TGX™ precast protein gels (BIO-RAD, 4561023) run in running buffer (25mM Tris, 192mM Glycine, 0.1% SDS) at 100 V for ~120 minutes. Transfer was performed using Trans-Blot Turbo Mini 0.2 µm PVDF Transfer Pack (BIO-RAD, 1704156) on a Trans-Blot Turbo transfer system (BIO-RAD) using the high molecular weight program. After transfer the membrane was washed in PBT (PBS + 0.1% Tween) several times and blocked with 5% milk powder in PBT (MPBT) at RT for 1 hour. The blots were incubated o/n at 4°C in 1º antibody (Ab290) in MPBT. The membranes were then washed several times in PBT and incubate in 2º antibody in MPBT at RT for 1 hour. Membranes were then washed several times in TBT and the signal was revealed using Clarify ECL substrate (BIO-RAD, 1705060) and imaged on a ChemiDoc XRS+ (BIO-RAD). The blots were then washed in PBT and blocked again in 5% MPBT for 1 hour at RT. They were then incubated o/n at 4°C with anti-actin antibody and processed as for the first antibody. Antibodies are listed in Supplementary Table 1

shRNA synthesis and injection. shRNAs were designed and synthesized as per the published protocol [6]. Briefly oligos (See supplementary table 2) were mixed to a final concentration of 20µM each in 20µl and then heated to 98°C for 5 minutes and then allowed to cool slowly to room temperature. 5.5 µl of this was then used as template in a 20 µl reaction with the AmpliScribe T7 transcription kit (Lucigen AS3107). Following

DNase treatment, the shRNA was purified using the RNA Clean and Concentrator Kit (Zymo, R1013). shRNAs were tested at a series of concentrations and 300 ng/µl was determined to be optimum in this case.

RTqPCR. For qPCR analysis animals were injected and allowed to develop for 4 hours and then RNA was extracted as for RNAseq analysis (see main Materials and Methods). . The SuperScript™ III first-strand synthesis system (Invitrogen, 18080051) was used to generate cDNA and it was primed using random hexamers (Custom oligos, Sigma). qPCR was performed using the Quantitect SYBR Green PCR mastermix (Qiagen, 204143) and ran on a BioRad CFX96 system. ATP synthetase was used as a reference gene and all oligos used are included in Supplementary Table 2.

Comparison between differentially expressed genes and metacell markers. Comparison was performed using custom in-house R script. Briefly, it takes N*ematostella* single cell atlas [7] data in excel format and the list of genes of interest to be compared to as an input and provides the heatmap as an output. It also converts 'v1g' gene models into 'NVE' (used in this study) and plots only those *NvLsd1*$^{-/-}$ genes which show two-fold or higher downregulation (log2 fold change <= -1) and which are present in at least one metacell. Color in the heatmaps correspond to the degree of downregulation (blue – the strongest, read – the weakest) in *NvLsd1* mutants versus controls at the indicated developmental stage.

Supplementary Figures and Tables

Supplementary Table 1: List of antibodies used in this study

Name	Company	Catalogue number	Concentration (IF)	Concentration (Western)
Rabbit anti-DsRed	Clontech	632496	1:100	
Mouse anti-mCherry	Clontech	632543	1:100	
Rabbit anti-GFP	Abcam	Ab290	1:200	1:20,000
Mouse anti-GFP	Abcam	Ab1218	1:200	
Goat anti-rabbit Alexa 488	Life Technologies	A11008	1:250	
Goat anti-rabbit Alexa 568	Life Technologies	A11011	1:250	
Goat anti-mouse Alexa 488	Life Technologies	A11001	1:250	
Goat anti-mouse Alexa 568	Life Technologies	A11004	1:250	
Rabbit anti-Actin	Abcam	A5060		1:1000
Goat Anti-Rabbit (HRP)	Abcam	Ab97051		1:10,000

Supplementary Table 2: List of primers used in this study

Name	Use	Sequence
*NvLsd1*_sgRNA1_Fw	Generating the template for the sgRNA used to generate the *NvLsd1* mutant	5' TAATACGACTCACTATAGGGTTTTGGCAACCTTAATAGTTT TAGAGCTAGAA 3'
*sgRNA*_Rv		5'AAAAGCACCGACTCGGTGCCACTTTTTCAAGTTGATAACGG ACTAGCCTTATTTTAACTTGCTATTTCTAGCTCTAAAAC 3'
*NvLsd1*_K/in_sgRNA1	Generating the sgRNAs used to generate the NvLsd1^{GFP} animals	5' TTCTAATACGACTCACTATAGGCATATATCTAGCGAGGTGTT TTAGAGCTAGA 3'
*NvLsd1*_K/in_sgRNA2		5' TTCTAATACGACTCACTATAGGATATATCTAGCGAGGTTGGTT TTAGAGCTAGA 3'
*NvLsd1*_Mut_Seq_Fw	PCR for sequencing the *NvLsd1* mutants	5' GCCGACGCCGTACTC 3'
*NvLsd1*_Mut_Seq_Rv		5' GCCTATAATGTACGAGCTGGTTTTGGC 3'
Repair_Template_Fw	Generating the repair template for the Knock-in	5' CGAAGTCGGGCGAGTGAGCCCACCCATGCCCCAACCTCGC GGCGGAGGAGGCTCAGG 3'
Repair_Template_Rv		5' GTTCGTCCGTGATACGCAGGGTCGGTGCAGCATATATCTAC TTGTCGTCATCGTCTTTGTAGTCCTTGTACAGCTCGTCCATGC3'
NvLsd1_F1	PCR to confirm the GFP insertion in NvLsd1^{GFP} animals (See Fig. S3)	5' CGGCTAACGTGGCACCAG 3'
NvLsd1_R1		5'GACAACTATTTCGTAGGCTCAAATTTCGCC 3'
NvLsd1_F2	To amplify the Lsd1^{GFP} cDNA (See Fig. S3)	5' ATGTCGATTCCACCATATCAAACTC 3'
NvLsd1_R2		5' CTAGCGAGGTTGGGGC 3'
NvLsd1_cDNA_Fw	To amplify *NvLsd1* coding sequence	5' ATGTCGATTCCACCATATCAAACTC 3'
NvLsd1_cDNA_Rv		5' CAGTTTCAGAGATTACAAGTTGAAAGGTTTTC 3'
NvLsd1_ATG_Fw_1HA	Amplify *NvLsd1* and add the 2HA tags	5' GACTACGCAGGTTACCCTTACGATGTTCCCGACTACGCAGG TTCCTCGATTCCACCATATCAAACTCCAATTCTACC 3'
NvLsd1_ATG_Fw_2HA		5'TAGATATCAGTTGGTGCTGAGTGCTCCACGATGTATCCCTAT GATGTTCCAGACTACGCAGGTTACCCTTACGATG 3'
NvLsd1_noSTOP_Rv		5' GCGAGGTTGGGGCATGG 3'
NvPOU4::mCherry_Fw1	Amplify the *NvPOU4:*:mCherry backbone with overhangs for *NvLsd1*	5' GCCCACCCATGCCCCAACCTCGCGCGGGCGGAGGTGGCA GCGTGAGCAAGGGCGAGGAAG 3'
NvPOU4::mCherry_Rv1		5' ATCGTAAGGGTAACCTGCGTAGTCTGGAACATCATAGGGAT ACATCGTGGAGCACTCAGCACCAAC 3'
NvH2B_Fwd	Amplify *NvH2B*	5' ATGCCTGCCAAGAAAAGAGCTCC3'
NvH2B_NoSTOP_Rv		5' TCCAGTGCTCGCTGAGTATTTGG 3'
NvPOU4::mCherry_Fw2	Amplify the *NvPOU4:*:mCherry backbone with overhangs for *NvH2B*	5'GCCGTCGCCAAATACTCAGCGAGCACTGGAGCGGGCGGCG GCGGCAGC3'
NvPOU4::mCherry_Rv2		5'CCCGGCAGGAGCTCTTTTCTTGGCAGGCATCGTGGAGCACT CAGCACCAAC 3'
*NvLsd1*_Internal_Fw1	Amplify the *NvPOU4*:NvLsd1-mCherry plasmid to insert the K644A mutation	5' GCACACACGTGGGGAGTTGTTTC 3'
*NvLsd1*_Internal_Rv1		5' GGAACTGCACAGCGGCAG 3'
*NvLsd1*_Internal_Fw2	Amplify the *NvPOU4*:NvLsd1-mCherry plasmid to insert the A520E mutation	5' CAGTGAGACATGTGCGCTACAGTAG 3'
*NvLsd1*_Internal_Rv2		5' CCAGCTCTTCTAATCTCTCGTCCAGC 3'
NvLsd1_ShRNA_Fw	Generating NvLsd1 shRNA	5' TAATACGACTCACTATAGATGAGGAGGAGGATGATATTCAA GAGATATCATCCTCCTCCTCATCTT 3'
NvLsd1_ShRNA_Rv		5'AAGATGAGGAGGAGGATGATATCTCTTGAATATCATCC TCCTCCTCATCTATAGTGAGTCGTATTA 3'

NvLsd1_Sram_Fw	Generating NvLsd1 shRNA	5’ TAATACGACTCACTATAGGAGGTGGAGAAGGTAATATTCAA GAGATATTACCTTCTCCACCTCCTT 3’
NvLsd1_Scram_Rv		5’ AAGGAGGTGGAGAAGGTAATATCTCTTGAATATTACCT TCTCCACCTCCTATAGTGAGTCGTATTA 3’
ATPsynt_se	ATP synthetase primers fro qPCR	5’ TGCTGGGAAAGTTCTGGACCAATG 3’
ATPsynt_as		5’ ACACCCTCCTTGACGGTAACATTC 3’
NvLsd1_se	NvLsd1 primers fro qPCR	5’ GCAAGAGGGCCAAAGTTGAGTAC 3’
NvLsd1_as		5’ CCTCCATAATCTCCTTGGGGATG 3’

Supplementary Figures

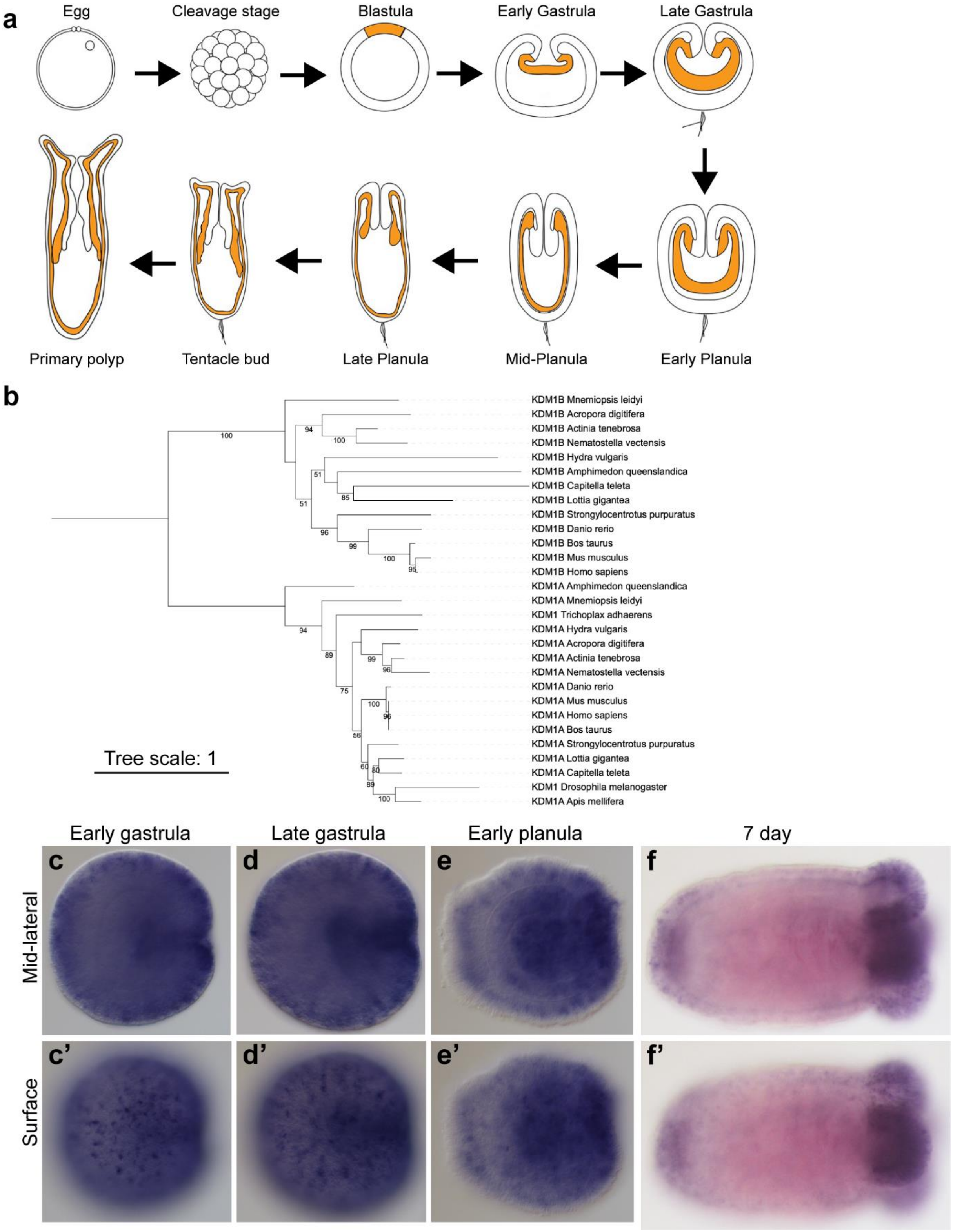

Supplementary Fig. 1: *Nematostella* life cycle and *NvLsd1* expression. (**a**) Schematic representation of *Nematostella* developmental stages shown throughout the paper. The endomesoderm is shown in orange. Adapted from [8]. (**b**) Phylogenetic

tree of Lsd1/KDM1 proteins based on AOD domains. Maximum-likelihood method was used to infer the tree and consensus tree was constructed from 1000 bootstrap trees. Numbers correspond to the bootstrap values and only values above 50 are shown.(**c-f**) RNA in-situ hybridization for *NvLsd1* during embryogenesis*.* Stage is shown on top. (**c-f**) show mid-lateral views and (c'-f') show surface views.

```
HsLSD1      MLSGKKAAAAAAAAAAAATGTEAGPGTAGGSENGSEVAAQPAGLSGPAEVGPGAVGERTP  60
NvLsd1      --------------------------------------------------------MSIP  4
                                                                       *

HsLSD1      RKKEPPRASPPGGLAEPPGSAG----------PQAGPTVVPGSATPMETGIAETPEGRRT  110
NvLsd1      PYQTPILPKPVGNFNVPQTSGQNSENQLYLADPKHGSHPSHKRLIDQKYLNEDAAGQRRT  64
              : *   .* *.:  *  *.             *: *             :    ::   ***

HsLSD1      SRRKRAKVEYREMDESLANLSEDEYYSEEERNAKAEKEKKLPPPPPQAPPEEENESEPEE  170
NvLsd1      SQRKRAKVEYTEVDEKLATLTDDELSDDDIPKEIMEAEEQQ-VENKFPEPEEEDEEEDDT  123
            *:******** *:**.**.*::**  .::  :   * *::           ****:*.* :

HsLSD1      PSGVEGAAFQSRLPHDRMTSQEAACFPDIISGPQQTQKVFLFIRNRTLQLWLDNPKIQLT  230
NvLsd1      PQGLEGAAFQSRLPFDKMTSQESSCFPDISQGPPHLQKQFLYIRNRVLQLWLENPQQQLT  183
            *.*:**********.*:*****::*****  .**  :  **  **:****.*****:**: ***

HsLSD1      FEATLQQLEAPYNSDTVLVHRVHSYLERHGLINFGIYKRIKPLPT-KKTGKVIIIGSGVS  289
NvLsd1      LEAAIPQIEPPNNSDLKLVQRVHAFLERYGSINFGVYKMAKMPPTLKKSPKVIIVGSGIA  243
            :**::  *:*  *  ***   **:***::***:*  ****:**   *   ** **:  ****:***::

HsLSD1      GLAAARQLQSFGMDVTLLEARDRVGGRVATFRKGNYVADLGAMVVTGLGGNPMAVVSKQV  349
NvLsd1      GLMAARQLQSFGIDVTMVEARERVGGRVATFRKGQYIADLGAMVLTGLGGNPLTVLNNQI  303
            ** *********:***::***:************:*:*******:*******::*:.:*:

HsLSD1      NMELAKIKQKCPLYEANG---------------------------QAVPKEKDEMVEQEF  382
NvLsd1      SMEVHKIRQKCPLYESLGKPIGRRHVQLTPRDHPWPSKSHIGASAQRVPKDKDEMVEREF  363
            .**:  **:*******:  *                            *  ***:******:**

HsLSD1      NRLLEATSYLSHQLDFNVLNNKPVSLGQALEVVIQLQEKHVKDEQIEHWKKIVKTQEELK  442
NvLsd1      NRLLEATSFLSHQLDFNYMNGKPVSLGHALELVIKMQEKQVKEQQVEHAKKILSLQDHMK  423
            ********:********  :*.******:***:**::.***:**::*:**  ***:.  *:.:*

HsLSD1      ELLNKMVNLKEKIKELHQQYKEASEVKPPRDITAEFLVKSKHRDLTALCKEYDELAETQG  502
NvLsd1      ENLAKMVSIQEKARQTHREYTEALKVKEPRDVTSEFLVKSKQRDLNALCREYDEYNEKQQ  483
            *  *  ***.::**  ::  *::*.**  :**  ***:*:*******:***.***:****   *.*

HsLSD1      KLEEKLQELEANPPSDVYLSSRDRQILDWHFANLEFANATPLSTLSLKHWDQDDDFEFTG  562
NvLsd1      MLDERLEELENNPPSDVYLSSRDRQILDWHFANLEFANATPLTALSLKHWDQDDDFEFSG  543
             *:*:*:*** *******************************::**************:*

HsLSD1      SHLTVRNGYSCVPVALAEGLDIKLNTAVRQVRYTASGCEVIAVNTR--STSQTFIYKCDA  620
NvLsd1      SHMTVRNGYSCLPAALAEGLDIRLNTAVRHVRYSRGGVEVVTQSTNKSSITTTQTFKADA  603
            **:*********:*.********:******:***:  .* **::  .*.   *  :  *   :*.**

                                                     *
HsLSD1      VLCTLPLGVLKQQPPAVQFVPPLPEWKTSAVQRMGFGNLNKVVLCFDRVFWDPSVNLFGH  680
NvLsd1      VLITLPLGVLKANPAAVQFHPPLPEWKMAAVHRMGFGNLNKVVLCFDRIFWDPNTNLFGH  663
            ** ********  :* **** *******  :**:****************:****..*****

HsLSD1      VGSTTASRGELFLFWNLYKAPILLALVAGEAAGIMENISDDVIVGRCLAILKGIFGSSAV  740
NvLsd1      VNGTTHTRGELFLFWNLYKAPVLISLVAGEAADNLENVPDDIIVSRAVGVLRGIFGASNV  723
            *..** :**************:*::*******.  :**:  **:**.*.:.:*:****:* *

HsLSD1      PQPKETVVSRWRADPWARGSYSYVAAGSSGNDYDLMAQPITPGPSI---PGAPQP--IPR  795
NvLsd1      PNPKESVVTRWKSDEWSRGSYSYVAAGSSGNDYDLMASPVAPLPTANVAPGTPQPLNPPR  783
            *:***:**:**::* *:*********************.*::* *:      **:***    **

HsLSD1      LFFAGEHTIRNYPATVHGALLSGLREAGRIADQFLGAMYTLPRQATPGVPAQQSPSM     852
NvLsd1      VFFAGEHTIRNYPATVHGALLSGLREAGRIADQFLGLPYEVGRVSPPMPQPR-----     835
            :************************************* : * : *     :
```

SWIRM domain

AOL domain

Tower domain

* Human K661 Nematostella 644

Supplementary Fig. 2: Alignment of human and *Nematostella* Lsd1 sequences

Alignment of the full-length NvLsd1 sequence with human LSD1. The SWIRM domain is highlighted in yellow, the Amine Oxidase-like (AOL) domain in green and the Tower domain in blue. The position of human lysine 661/ *Nematostella* lysine 644 is marked with a red asterisk. The alignment was performed using Clustal Omega [9].

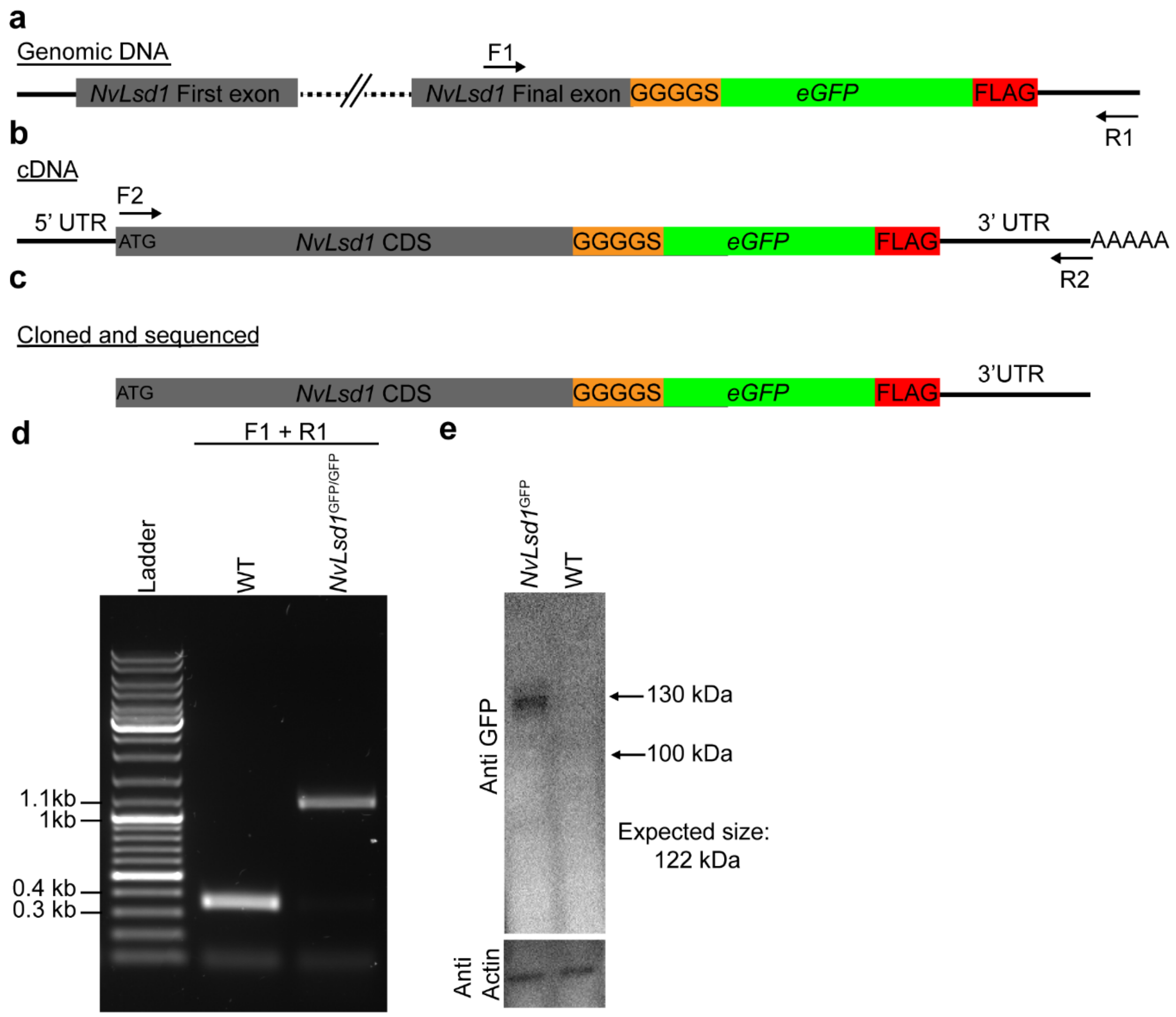

Supplementary Fig.3: Confirmation of *GFP* knock-in into the *NvLsd1* locus.

(**a**) Schematic showing the insertion of *eGFP* into the genome in frame with *NvLsd1* and indicating the position for the primers used to verify the insertion. (**b**) Schematic of the predicted full length *NvLsd1*^{GFP} cDNA with the inserted sequences. F2 and R2 indicate the primers used to amplify and clone the full-length coding sequence and 3'UTR from *NvLsd1*^{GFP} animals. (**c**) Schematic of the portion of *NvLsd1*^{GFP} cDNA which was cloned and sequenced. (**d**) Agarose gel of a PCR on genomic DNA from WT and homozygous *NvLsd1*^{GFP} animals showing the insertion of a single copy of GFP into the *NvLsd1* locus. The primers used are indicated on top. The expected size of the band from wildtype (WT) is 336 bps and from *NvLsd1*^{GFP} animals is 1089 bps. The band in

the *NvLsd1*^{GFP} lane was excised and sequenced to confirm it is the correct sequence. (**e**) Western blot on protein from *NvLsd1*^{GFP} and wild-type (WT) animals. The blot was probed for GFP (shown on top) and Actin (shown on the bottom). Sizes on the right indicate the positions of the ladder. The expected size of the NvLsd1-GFP fusion protein is 122 kDa. Experiments in (d) and (e) were repeated twice with the same result.

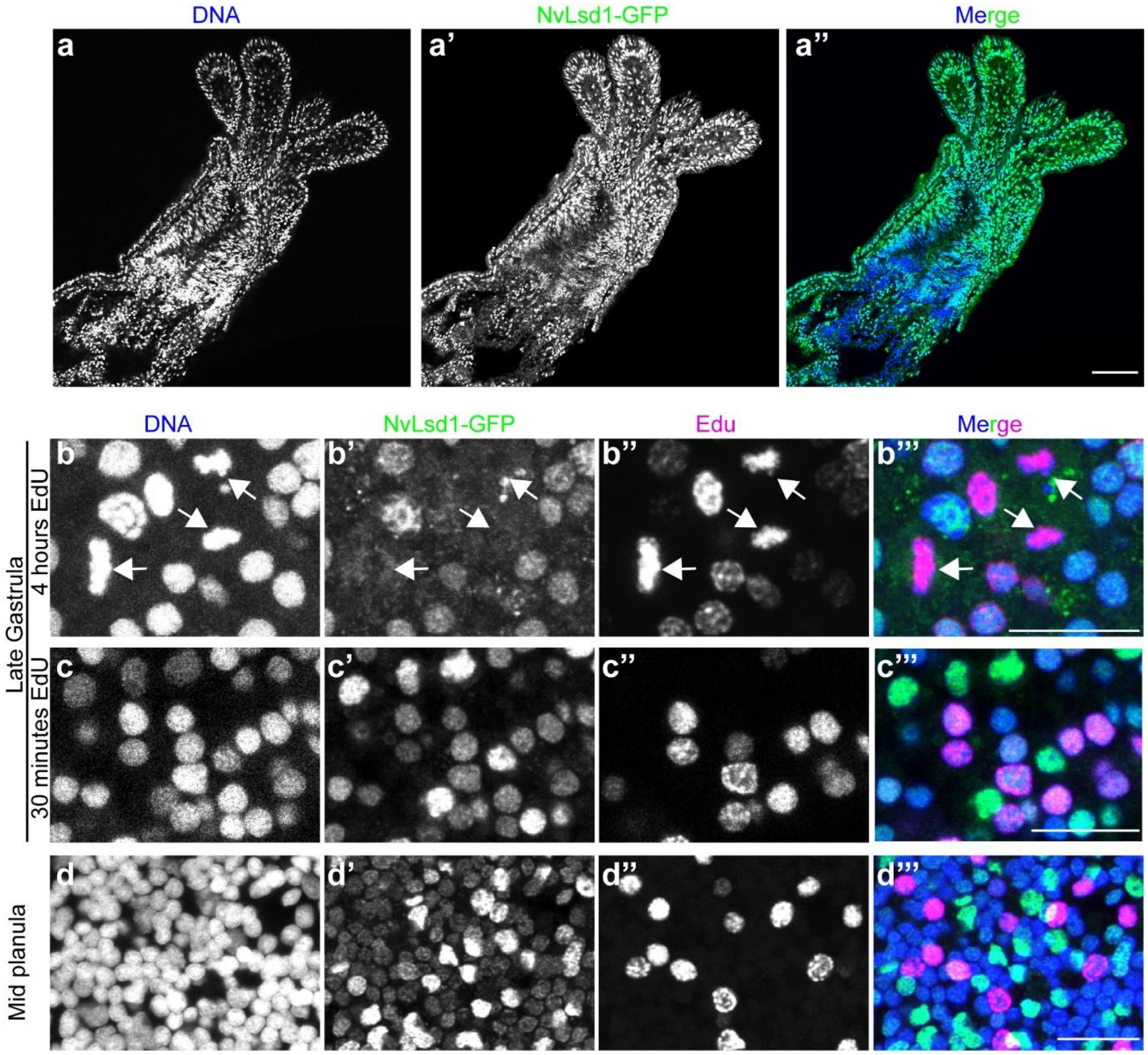

Supplementary Fig.4: Additional EdU labelling and primary polyp expression in *NvLsd1*^{GFP} animals.

(**a**) Confocal images of immunofluorescence staining on *NvLsd1*^{GFP} primary polyps. (**b-d**) Confocal images of immunostaining along with Click-iT-Edu detection showing close ups of the ectoderm of a late gastrula incubated with EdU for 4 hours (**b**), late gastrula incubated with EdU for 30 minutes (**c**) and mid-planula incubated with EdU for 30 minutes (**d**). Stage is shown on the left. Arrows in (**b**) point to mitotic nuclei. ClickIT-EdU is shown in magenta, Lsd1-GFP in green and DNA in blue. Images shown are

representative samples from at least two independent replicates with >10 animals per replicate. Scale bars: 50 μm (a), 10 μm (b-d)

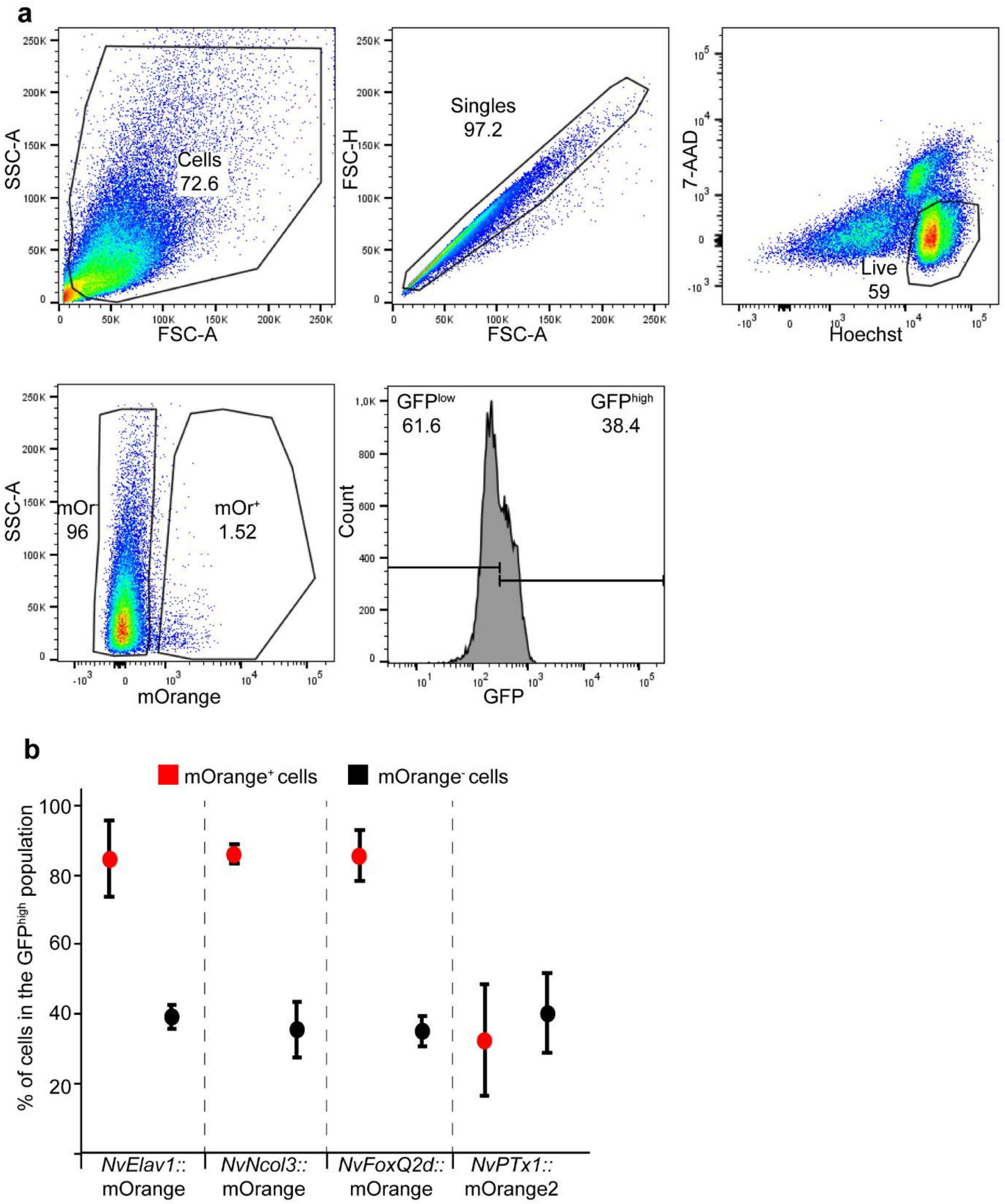

Supplementary Fig. 5: Flow cytometry analysis of *NvLsd*^{GFP} animals

(**a**) FlowJo plots showing an example of the gating strategy used to analyze the flow cytometry data. (**b**) Plots showing the percentage of mOrange^{+} (red) and mOrange^{-} (black) cells that fall within the GFP^{high} gate. The data shows the average of 3

independent replicates and the error bars show the standard deviation centered on the mean.

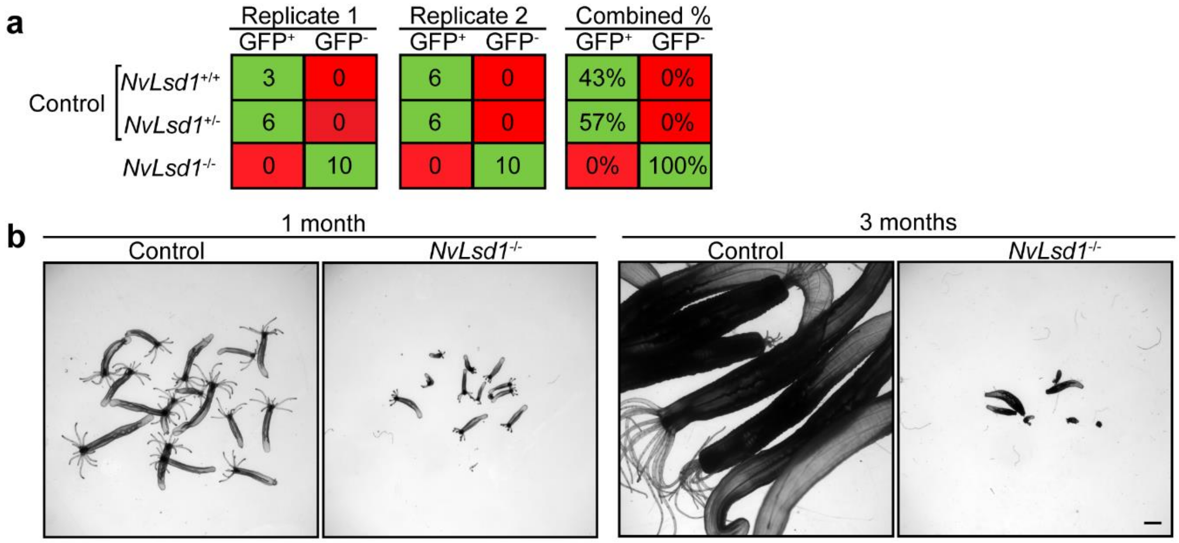

Supplementary Fig. 6: Further characterization of *NvLsd1* mutants

(**a**) Genotyping of GFP^{+} and GFP^{-} animals from the crosses shown in Figure 3A. (**b**) Images of animals shown in Figure 3f-i after 1 month and 3 months. Scale bar: 1 mm

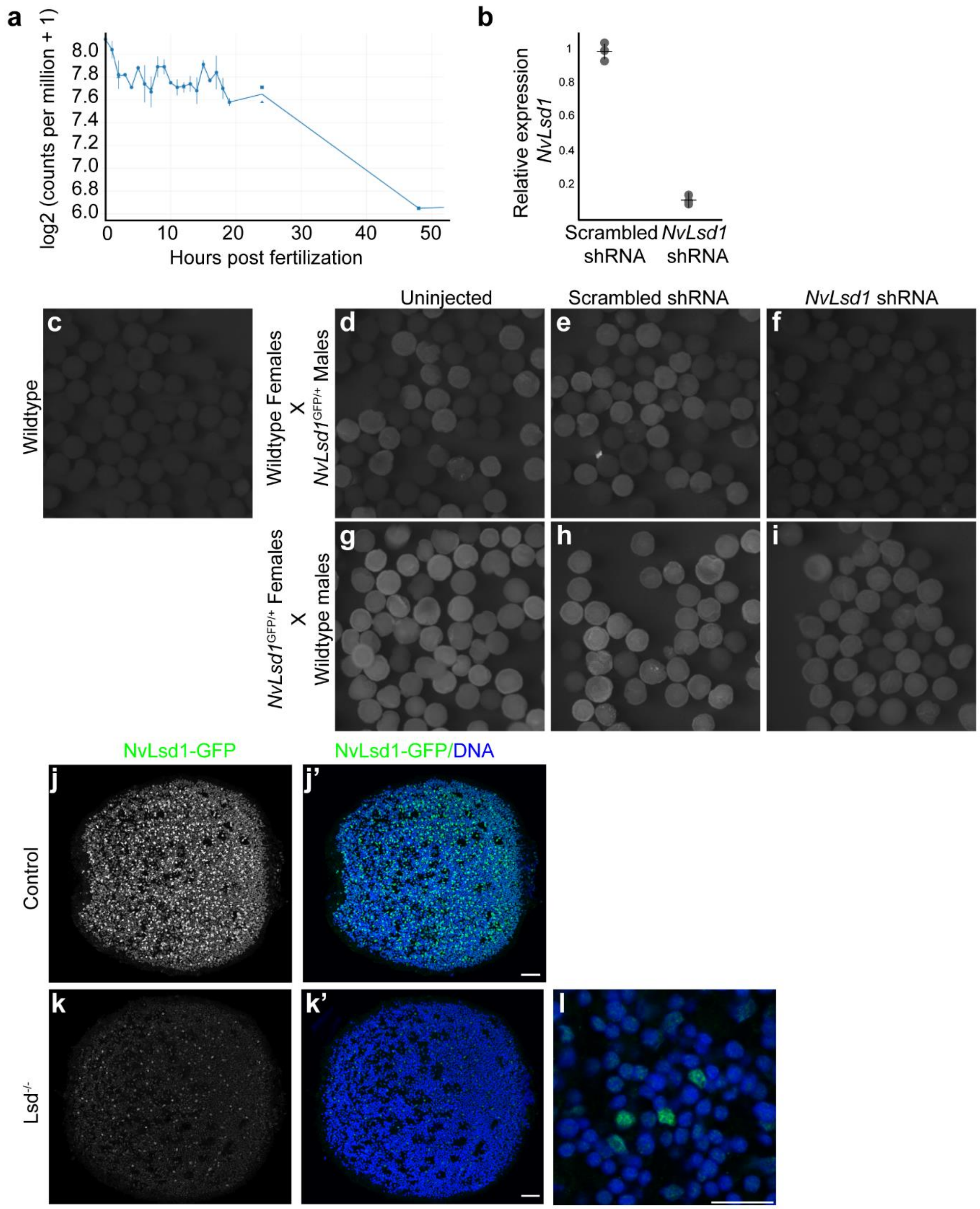

Supplementary Fig. 7: *NvLsd1* is a maternally deposited gene.

(**a**) RNAseq data from the NrERTx database [10] shows that *NvLsd1* mRNA is maternally deposited and high in early embryos. Error bars represent standard error of the mean of 2 replicates. (**b**) RTqPCR of embryos six hours post fertilization (approximately 4 hours post injection) showing relative levels of *NvLsd1* in scrambled control or *NvLsd1*

shRNA injected embryos. Chart shows data from 3 biological replicates and each replicate is represented by a dot. The center line represents the mean. (**c-i**) images of wildtype animals (c) or transgenic embryos (d-i) from shRNA injections. The crosses used to generate the embryos are indicated on the left and the treatment in indicated on top. This was repeated two times, independently. (**j-l**) Anti-GFP immunostaining in control (j) or *NvLsd1*$^{-/-}$ (k, l) mid-planula. (l) shows a close up taken at a higher detector threshold. Images shown are representative samples from at least two independent replicates with >10 animals per replicate. Scale bars: 25 µm (j, k) and 10 µm (l).

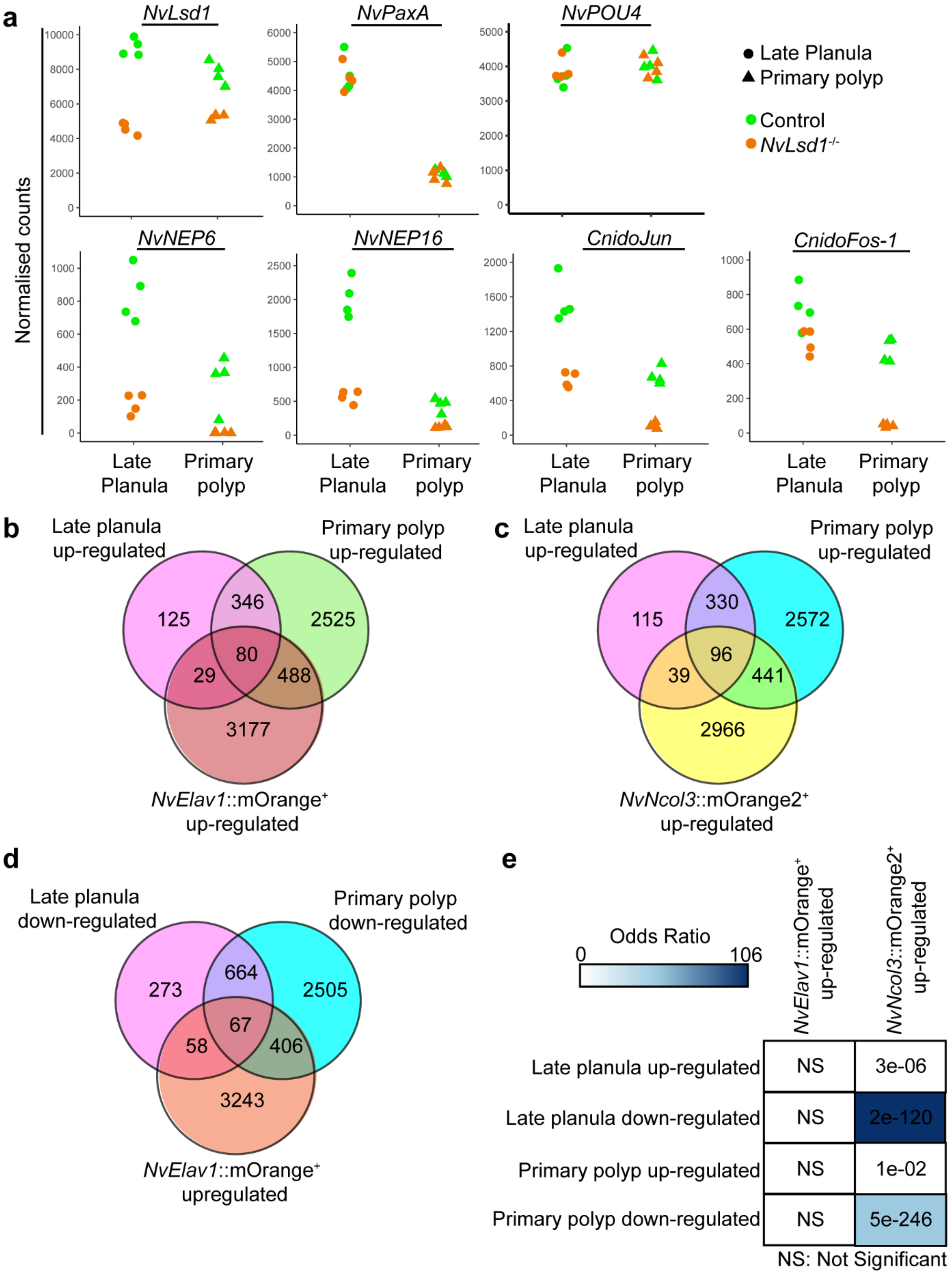

Supplementary Fig. 8: Comparison of differentially expressed genes in *NvLsd1* mutant with neural transcriptomes.

(**a**) Plots showing normalized count data for selected genes in *NvLsd1* mutants. Control samples are shown in green and mutants in orange. Late planula samples are represented by circles and primary polyp samples are shown as triangles. (**b**, **c**) Venn diagram comparing genes up-regulated in *NvLsd1*$^{-/-}$ late planula and primary polyp with genes up-regulated in *NvElav1*::mOrange^{+} (B) and *NvNcol3*::mOrange2^{+} (c) cells.

(**d**) Venn diagram comparing genes down-regulated in *NvLsd1*$^{-/-}$ late planula and primary polyp with genes upregulated in *NvElav1*::mOrange^{+} cells. (**e**) Comparison of the overlap between up- and down-regulated genes in *NvLsd1*$^{-/-}$ late planula and primary polyps with those up-regulated in *NvNcol3*::mOrange2^{+} and *NvElav1*::mOrange^{+} cells using the GeneOverlap R package using genes with a log2 fold change threshold of 1. The strength of the blue color indicates the odds ratio and numbers indicated the p-value, calculated using one-sided Fisher's exact test.

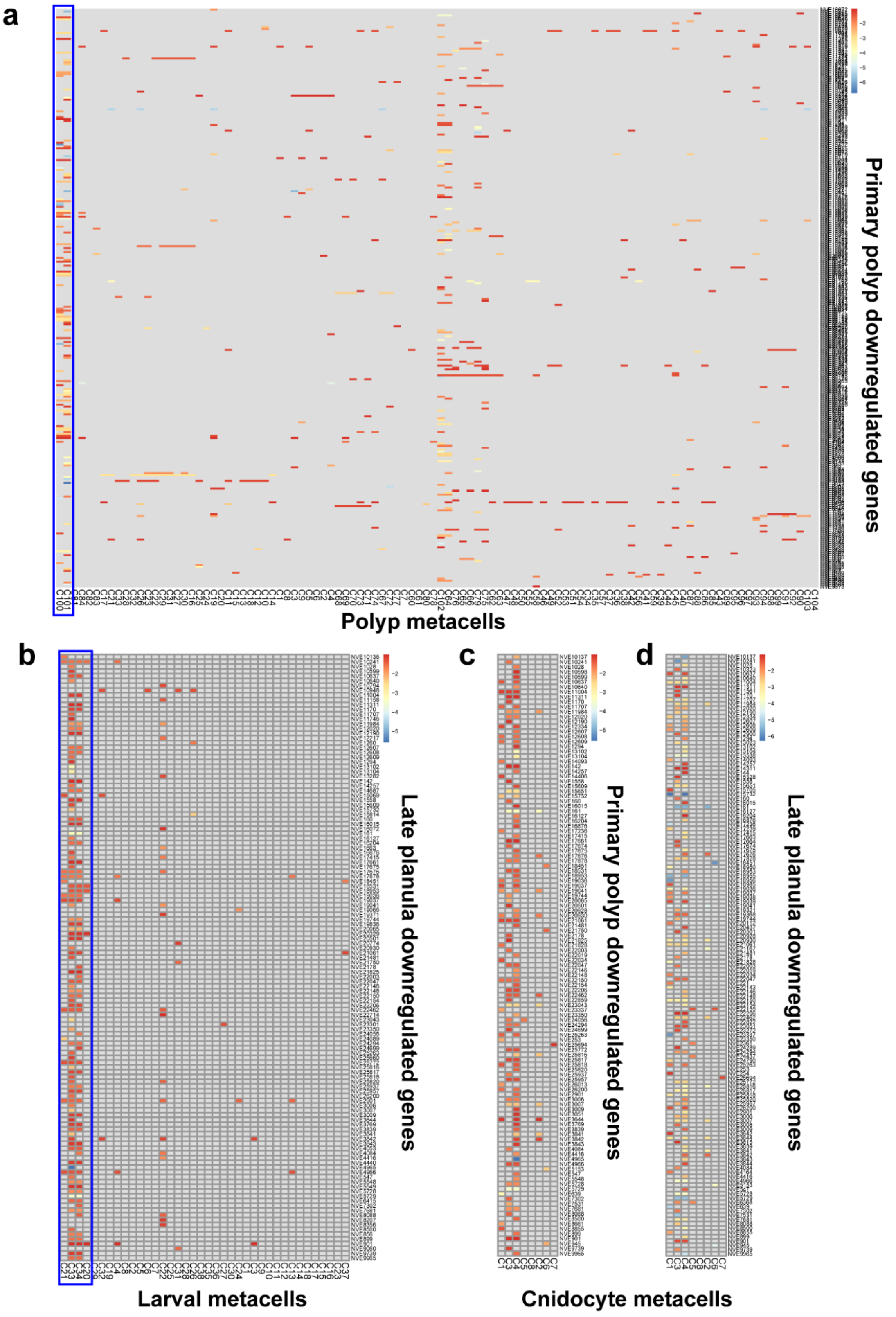

Supplementary Fig. 9. Comparison of genes differentially expressed in *NvLsd1* mutants to the *Nematostella* single cell atlas.

Heatmaps show enrichment of the genes downregulated in *NvLsd1* mutants in specific metacells from the *Nematostella* single cell atlas. Row names of the heatmaps are gene names and column names are metacell names**.** (**a**) Comparison of genes downregulated at primary polyp stage to aduly polyp metacells**.** Primary polyp *NvLsd1*$^{-/-}$ downregulated genes are enriched in metacells corresponding to cnidocytes (indicated by a blue box.). (**b**) Comparison of genes downregulated at late planula stage to larval metacells**.** Late planula *NvLsd1*$^{-/-}$ downregulated genes show enrichment in metacells corresponding to cnidocytes (indicated by a blue box). (**c**, **d**) Genes downregulated at either primary polyp (c) or late planula (d) stages compared to the cnidocyte metacells show a strong over representation in the more differentiated cnidocyte metacells (to the left of the heatmap). Only genes downregulated two-fold or more are shown (log2 fold change <= -1). Color in the heatmaps correspond to the degree of downregulation (blue – the strongest, read – the weakest).

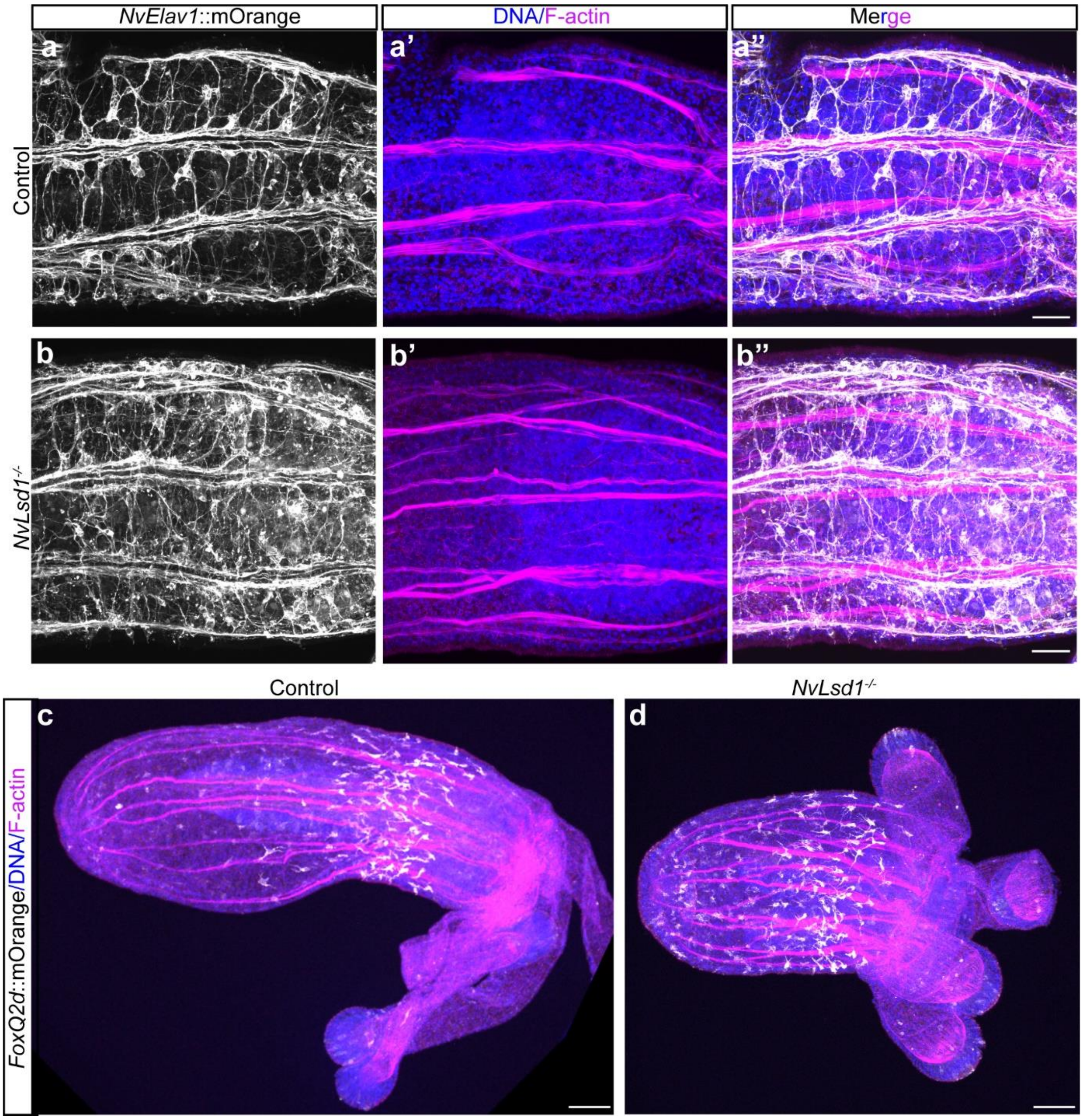

Supplementary Fig. 10: Analysis of *NvElav1*::mOrange and *NvFoxQ2d*::mOrange transgenes in *NvLsd1* mutants.

(**a-d**) Confocal images of immunofluorescence staining on control and *NvLsd1*$^{-/-}$ primary polyps in the background of the *NvElav1*::mOrange (**a, b**) *NvFoxQ2d*::mOrange (**c, d**) transgenes. (**a, b**) show close ups of the endoderm and (**c, d**) show full primary polyps. mOrange in shown in gray, DNA in blue and F-actin in magenta. Images shown are representative samples from at least two independent replicates with >10 animals per replicate. Scale bars: 20 µm (a, b) and 50 µm (c, d).

Supplementary references

1. Mistry, J. *et al.* Pfam: The protein families database in 2021. *Nucleic Acids Res* **49**, D412-D419 (2021).
2. Katoh, K., Misawa, K., Kuma, K. & Miyata, T. MAFFT: a novel method for rapid multiple sequence alignment based on fast Fourier transform. *Nucleic Acids Res* **30**, 3059-3066 (2002).
3. Nguyen, L.T., Schmidt, H.A., von Haeseler, A. & Minh, B.Q. IQ-TREE: a fast and effective stochastic algorithm for estimating maximum-likelihood phylogenies. *Mol Biol Evol* **32**, 268-274 (2015).
4. Richards, G.S. & Rentzsch, F. Transgenic analysis of a SoxB gene reveals neural progenitor cells in the cnidarian Nematostella vectensis. *Development* **141**, 4681-4689 (2014).
5. Tourniere, O. *et al.* NvPOU4/Brain3 Functions as a Terminal Selector Gene in the Nervous System of the Cnidarian Nematostella vectensis. *Cell Rep* **30**, 4473-4489 e4475 (2020).
6. Karabulut, A., He, S., Chen, C.Y., McKinney, S.A. & Gibson, M.C. Electroporation of short hairpin RNAs for rapid and efficient gene knockdown in the starlet sea anemone, Nematostella vectensis. *Dev Biol* **448**, 7-15 (2019).
7. Sebe-Pedros, A. *et al.* Cnidarian Cell Type Diversity and Regulation Revealed by Whole-Organism Single-Cell RNA-Seq. *Cell* **173**, 1520-1534 e1520 (2018).
8. Layden, M.J., Rentzsch, F. & Rottinger, E. The rise of the starlet sea anemone Nematostella vectensis as a model system to investigate development and regeneration. *Wiley Interdiscip Rev Dev Biol* **5**, 408-428 (2016).
9. Madeira, F. *et al.* The EMBL-EBI search and sequence analysis tools APIs in 2019. *Nucleic Acids Res* **47**, W636-W641 (2019).
10. Warner, J.F. *et al.* NvERTx: a gene expression database to compare embryogenesis and regeneration in the sea anemone Nematostella vectensis. *Development* **145** (2018).
