# Supplementary material for: Histone demethylase Lsd1 is required for the differentiation of neural cells in Nematostella vectensis
Source: Nat Commun. 2022 Jan 24;13:465. doi: 10.1038/s41467-022-28107-z (PMC8786827; doi:10.1038/s41467-022-28107-z)
Supplement: Supplementary file 3 — Description of Additional Supplementary Files [file 41467_2022_28107_MOESM3_ESM.pdf]

Description of Additional Supplementary Files

Supplementary Data 1: **Differentially expressed genes in *NvLsd1* mutants vs controls.** Given are the lists of genes up-regulated (upreg) or down-regulated (downreg) at late planula (planula) and primary polyp (polyp) stages using either no fold change cut off [Non-stringent (Non-S)] or a log2 fold cut off of 1 [Stringent(S)].

Supplementary Data 2: Genes upregulated in *NvNcol3*::mOrange2$^+$ and *NvElav1*::mOrange$^+$ cells. Given are the lists of genes up-regulated (upreg) in *NvNcol3*::mOrange2$^+$ (Ncol) and *NvElav1*::mOrange$^+$ (Elav) cells compared to mOrange$^-$ cells. Lists of genes generated using no fold change cut off [Non-stringent(Non-S)] or a log2 fold cut off of 1 [Stringent(S)] are given.

Supplementary Data 3: List of sequences and accession codes for proteins used to generate the Phylogeny in Supplementary Fig. 1b.

Supplementary Data 4: Alignment file for the Phylogeny in Supplementary Fig. 1b.
